# Regional variations in transepidermal water loss, eccrine sweat gland density, sweat secretion rates and electrolyte composition in resting and exercising humans

**DOI:** 10.1186/2046-7648-2-4

**Published:** 2013-02-01

**Authors:** Nigel AS Taylor, Christiano A Machado-Moreira

**Affiliations:** 1 Centre for Human and Applied Physiology, School of Health Sciences, University of Wollongong, Wollongong, New South Wales, 2522, Australia

**Keywords:** Eccrine sweat gland, Electrolyte, Glandular density, Insensible perspiration, Sudomotor, Sweat, Thermal sweating, Transepidermal water loss

## Abstract

Literature from the past 168 years has been filtered to provide a unified summary of the regional distribution of cutaneous water and electrolyte losses. The former occurs via transepidermal water vapour diffusion and secretion from the eccrine sweat glands. Daily insensible water losses for a standardised individual (surface area 1.8 m^2^) will be 0.6–2.3 L, with the hands (80–160 g.h^−1^) and feet (50–150 g.h^−1^) losing the most, the head and neck losing intermediate amounts (40–75 g.h^−1^) and all remaining sites losing 15–60 g.h^−1^. Whilst sweat gland densities vary widely across the skin surface, this same individual would possess some 2.03 million functional glands, with the highest density on the volar surfaces of the fingers (530 glands.cm^−2^) and the lowest on the upper lip (16 glands.cm^−2^). During passive heating that results in a resting whole-body sweat rate of approximately 0.4 L.min^−1^, the forehead (0.99 mg.cm^−2^.min^−1^), dorsal fingers (0.62 mg.cm^−2^.min^−1^) and upper back (0.59 mg.cm^−2^.min^−1^) would display the highest sweat flows, whilst the medial thighs and anterior legs will secrete the least (both 0.12 mg.cm^−2^.min^−1^). Since sweat glands selectively reabsorb electrolytes, the sodium and chloride composition of discharged sweat varies with secretion rate. Across whole-body sweat rates from 0.72 to 3.65 mg.cm^−2^.min^−1^, sodium losses of 26.5–49.7 mmol.L^−1^ could be expected, with the corresponding chloride loss being 26.8–36.7 mmol.L^−1^. Nevertheless, there can be threefold differences in electrolyte losses across skin regions. When exercising in the heat, local sweat rates increase dramatically, with regional glandular flows becoming more homogeneous. However, intra-regional evaporative potential remains proportional to each local surface area. Thus, there is little evidence that regional sudomotor variations reflect an hierarchical distribution of sweating either at rest or during exercise.

## Review

### Introduction

Human skin contains glands that secrete watery fluids directly onto the skin surface: apocrine glands (milky fluid), eccrine sweat glands (serous fluid) and the apoeccrine glands (serous fluid [1]). The focus of this review is upon the eccrine sweat glands, with a particular emphasis upon the thermal sweating that subserves temperature regulation at rest and during exercise. However, brief discussion of non-thermal control is included, and for completeness, the movement of water through the skin in both its liquid (active sweating) and gaseous phases (transepidermal water loss), as well as the electrolyte content of sweat, are covered.

The classical works of Kuno [2,3] provided comprehensive summaries of sweat gland function, to which significant supplementary contributions have been provided by List [4], Weiner and Hellmann [5], Wang [6], Sato [7], Quinton [8] and Sato et al. [9]. However, this area of research is relatively small, and advances have frequently been dictated by the growth and decline of a few laboratories, often driven by medical, military or commercial needs. Recently, this field has experienced another resurgence, with one stimulus coming from clothing and fabric manufacturers seeking to develop garments that optimise evaporative heat dissipation. The vapour resistance of these textiles and ensembles is evaluated using thermal manikins, and so the development of the latest generation of these devices demands precise information concerning the regional distribution of eccrine sweat glands and their rates of secretion [10].

Two groups recently revisited the regional distribution of human sweating (Loughborough University, UK and the University of Wollongong, Australia). The former was primarily focussed on the requirements of clothing manufacturers, whilst the latter addressed questions relevant to the design of sweating, thermal manikins. Both assumed that the literature might provide these answers, but precise details regarding the topography of sweating were missing. Accordingly, both laboratories independently embarked upon comprehensive projects to obtain this information, and in the contributions that follow, the classical and the most recent mapping data are combined, analysed and critically reviewed to provide descriptions of the regional variations in human eccrine sweat gland density and local sweat secretion rates during the thermal loading of healthy, resting and exercising individuals. To these data are added updates on the regional distributions of transepidermal water loss and variations in the composition of sweat. To the best of our knowledge, this comprehensive combination of information is not currently available.

#### A brief historical background

Plants and animals lose water passively through semi-permeable membranes, even under cool conditions. In humans, this transepidermal water vapour loss was recognised by the ancient Greeks [11], but not understood. In 1614, the Italian physiologist Santorio Sanctorius (1561–1636) quantified changes in body mass due to this perspiration [12], which occurs by osmotic diffusion through the epidermis [13]. However, separate and independent water losses can occur through the activation of sweat glands when exposed to thermal [13], psychogenic [3,14,15] and exercise stresses [16].

The first identification of the eccrine sweat pores is often attributed to the Italian physiologist Marcello Malpighi (1628–1694, [2]), although Empedocles (495–435 BCE) was certainly aware of their existence some 2,000 years earlier [11], and the English microscopist Nehemiah Grew (1641–1712) described the epidermal ridges and sweat pores of the hands and feet in 1684 [17]. Indeed, his text also shows an awareness of the differences in sweat secretion from the glabrous (hairless) and non-glabrous (hairy) surfaces of these appendages, and the Dutch microbiologist Antonie van Leeuwenhoek (1632–1723) also wrote about sweating [18] and the sweat pores of the hands [19]. However, the precise control of sweat glands from these glabrous surfaces has, until recently, remained unresolved [15,20,21].

The sweat glands themselves were discovered in 1833 by the Czech physiologist Johannes Purkinjé (1787–1869), with their description provided by Wendt [22], one of his students. Within a decade, the German anatomist Karl Krause (1797–1868) undertook the first recorded evaluation of regional sweat gland densities [23]. Then, the French histologist Louis-Antoine Ranvier (1835–1922) grouped the secretory glands of the skin into two classes on the basis of their mode of secretion [24]: the holocrine glands (sebaceous and meibomian glands) and the merocrine glands (sweat glands). Some 20–30 years later, the merocrine classification was subdivided into apocrine and eccrine sweat glands [25,26], with Sato et al. [1] eventually adding a third class; the apoeccrine glands. For this review, the primary focus is upon the eccrine glands.

#### Transepidermal water loss

While transepidermal water loss (insensible perspiration) is not a principal emphasis, it is necessary to consider water loss in both its gaseous and liquid states to gain a more complete evaluation of water movement across the skin surfaces. Pinson [13] established that this form of water loss does not involve the sweat glands. Instead, vapour diffuses through the largely impermeable epidermis (transpiration), down the vapour pressure gradient within the *stratum corneum*[27] and into the boundary layer of air. Indeed, this semi-permeable protective barrier encases the moisture-laden tissues of the body, and in so doing, it participates in fluid homeostasis [28,29].

This water loss is imperceptible, and so it was originally described by Sanctorius [30] as insensible perspiration. It includes water lost from the cutaneous and pulmonary surfaces, but excludes losses associated with the neural (autonomic) activation of sweat glands [13,31], although unstimulated sweat glands do provide conduits for this transpirational loss. This distinction is most important, for one can, in extremely dry conditions, recruit quite high sweat flows whilst remaining totally dry at the skin surface, and unaware to this fluid loss [31,32]. Thus, while insensible perspiration remains the name of choice for some, its ambiguity has seen it replaced by transepidermal water loss, which describes passive vapour diffusion through the epidermis.

In resting, thermoneutral individuals, whole-body water loss is widely accepted to occur at about 30 g.h^−1^ in adult males [33], with approximately 50% of this passing through the skin [34]; transepidermal loss. This gradual water flux is dependent, to a slight extent, upon the thickness of the stratum corneum (being smaller for thicker tissue layers within each region [35,36]), the size of the intervening corneocytes (inverse relationship [29]), local tissue temperature (greater when warmed [37-39]), the boundary-layer water vapour pressure (greater at lower vapour pressures [13,39,40]) and even posture (greater when upright [33]). However, it is not influenced by changes in cutaneous blood flow, unless there is a corresponding elevation in local tissue temperature [13]. Among skin regions, the thickness of the stratum corneum is remarkably consistent (10–20 μm), with notable exceptions being evident at the hands and feet (both 400–600 μm [27,41]), although these sites do not have lower water diffusion constants. Indeed, the steady-state water flux through the skin from the abdomen is about 10% of that observed from the plantar surface of the foot and 30% of that from the palm [27].

**Figure 1:**
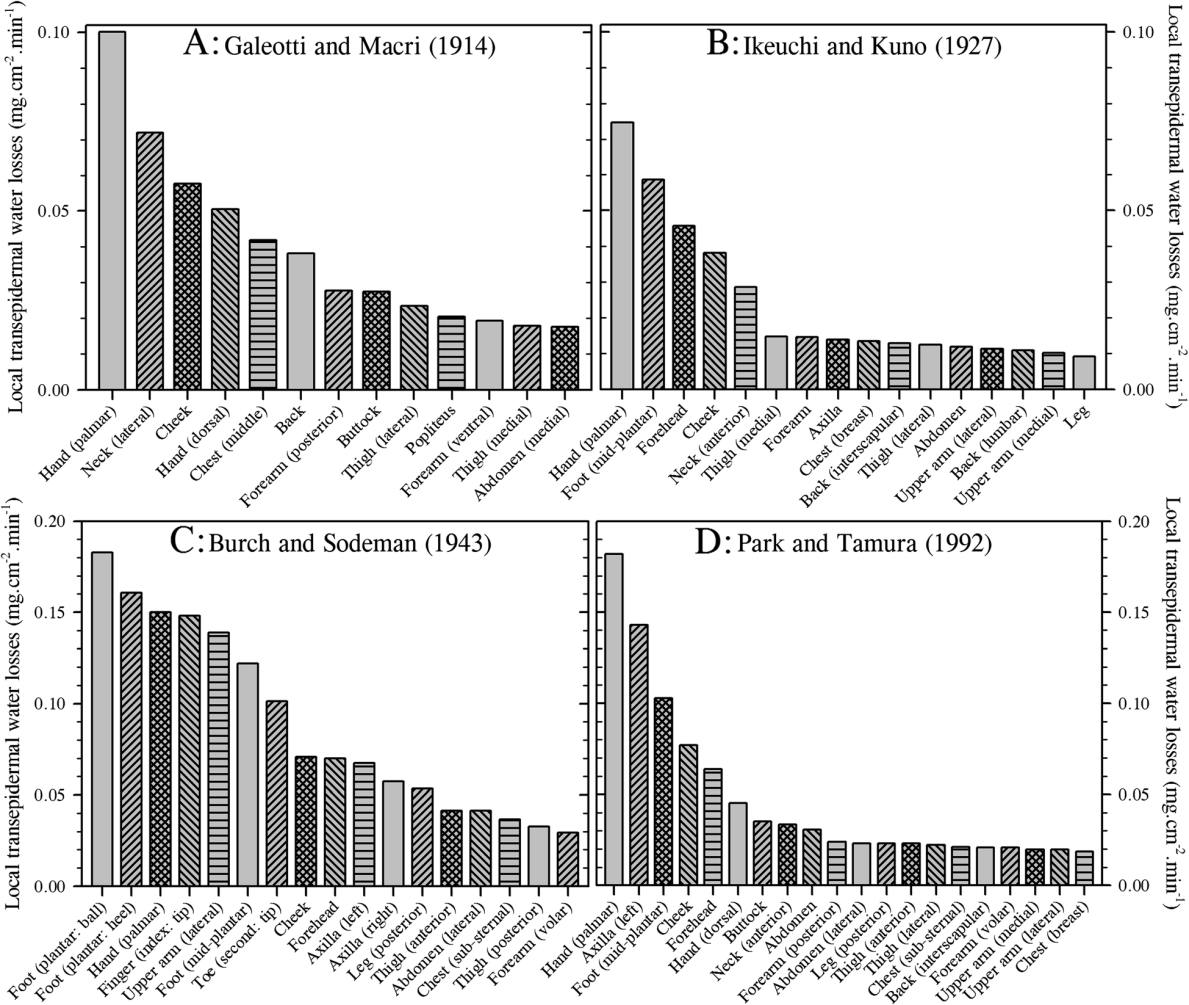
**Regional variations in transepidermal water loss.** Data were extracted from four studies: (**A**) Galeotti and Macri [42]. (**B**) Ikeuchi and Kuno [43]. (**C**) Burch and Sodeman [44]. (**D**) Park and Tamura [45]. Data are reported in identical units and arranged in descending order. To highlight differences among sites within each study, two ordinate scales have been used. Nine sites from Park and Tamura [45] have been excluded for simplicity, leaving only the sites in common with the other reports.

It is of particular interest in the current context to note that transepidermal water loss is not uniform over the body surface, and this variability is most evident at the hands and feet. Indeed, four groups have elegantly demonstrated this fact (Figure 1): Galeotti and Macri [42] (13 sites), Ikeuchi and Kuno [43] (16 sites), Burch and Sodeman [44] (17 sites) and Park and Tamura [45] (20 sites). Although each reported qualitatively similar patterns of water loss, with mean losses from the hands and feet occurring at two to four times that from the other surfaces, the absolute values from these studies varied considerably across both subjects and experiments. In each study, small chambers were placed over the target skin sites to collect water vapour, the losses of which were determined either gravimetrically or using hygrometry [45]. In the former methods, mass changes were recorded using an hygroscopic salt (calcium chloride [42]), filter papers [43] or water-vapour condensation (dry oxygen, [44] after [46]). In the oldest of these studies, Galeotti and Macri [42] measured water loss in five subjects, resting under cool to thermoneutral conditions (15.5°C–23°C). Ikeuchi and Kuno [43] collected data from eight participants (four males and four females) studied under thermoneutral conditions (22.8°C–26.0°C). Burch and Sodeman [44], and subsequently Sodeman and Burch [47], measured water loss during supine, thermoneutral rest (23°C) in 46 individuals (32 males and 14 females). Finally, Park and Tamura [45] studied ten resting women (prone and supine) at 25°C.

It is evident from Figure 1 that the observations of Burch and Sodeman [44] and values for the hand and axilla from Park and Tamura [45] are almost twofold greater than those reported by the other groups. Indeed, if one disregards duplicate measures from the same segment within each study, then the mean whole-body transepidermal water loss rates from these investigations are 0.04 mg.cm^−2^.min^−1^[42], 0.02 mg.cm^−2^.min^−1^[43], 0.07 mg.cm^−2^.min^−1^[44] and 0.04 mg.cm^−2^.min^−1^[45]. These equate with respective flows of 42.7, 25.7, 75.9 and 43.4 g.h^−1^, assuming a constant body surface area of 1.8 m^2^. Of these, only the second approximates the classical value reported by Benedict and Wardlaw [33] (15 g.h^−1^), as determined from whole-body mass changes. More recent data from six sites, but only three body segments (forehead, forearm, abdomen), are also supportive of these higher values, averaging 0.03 mg.cm^−2^.min^−1^ or 28.9 g.h^−1^[48].

However, one must cautiously avoid the assumption that consensual values are inherently more correct. Inspection of data collected by Ikeuchi and Kuno [43] and Burch and Sodeman [44] from the same sites reveals considerable inter-subject variability as well as overlapping values for three of the seven common sites. Furthermore, each group used a slightly different technique to collect water vapour. It may be said that the method of Ikeuchi and Kuno [43] involved the passive accumulation of water vapour, and one might expect these data to more closely correspond with those derived from mass changes. On the other hand, Galeotti and Macri [42], Burch and Sodeman [44] and Park and Tamura [45] employed techniques that optimised vapour flux. Such methods, particularly those of Burch and Sodeman [44], present an ideal water vapour pressure gradient for the movement of water molecules through the epidermis, with the skin and its boundary layer being kept drier than would normally be experienced. Thus, whilst it may be suggested that such a method may tend to exaggerate water loss in less-than-ideal circumstances, it may equally be noted that, without this state, the vapour pressure gradient would continually fall and thereby impede water loss over time. Accordingly, it is recommended that the data of Burch and Sodeman [44] be treated as ideal for naked skin, whilst those of Ikeuchi and Kuno [43] may represent water losses for fully clothed states in which the boundary-layer water vapour pressure gradually approaches saturation. In this way, one may consider data from these two studies to represent the upper and lower ranges, with values from Galeotti and Macri [42] and Park and Tamura [45] falling within these limits.

It is therefore concluded that whole-body, transepidermal water loss ranges between 0.02 and 0.07 mg.cm^−2^.min^−1^, or 26–43 g.h^−1^. These values equate with daily losses of 0.6–2.3 L for an individual of 1.8 m^2^. The hands and feet stand out as sites of considerable vapour loss, with the hands losing between 80 and 160 g.h^−1^ and the feet between 50 and 150 g.h^−1^, with only Burch and Sodeman [44] reporting this loss to be greater at the foot. Sites around the head and neck appear to experience intermediate losses (40–75 g.h^−1^), with all remaining sites being uniformly low (15–60 g.h^−1^), as originally described by Kuno [2]. We shall now consider active (autonomically mediated) water loss through the skin via the eccrine sweat glands.

### Regional variations in eccrine sweat gland density

#### The structure and development of eccrine glands

Eccrine sweat glands have a mass of about 30–40 μg, are found within the first 3 mm of the skin [7] and appear over the entire body surface. These structures develop within the stratum germinativum (the layer of keratinocytes at the base of the epidermis) and begin to appear beyond 12–13 weeks of gestation [49,50]. They grow down through the dermis and upwards in a helical path through the epidermis [51,52] before penetrating the skin as a sweat pore. Their embryonic development is essentially complete after 22 weeks of gestation [49], with glands being visible beyond 32 weeks [53].

Secretory coils of these glands exist within the dermis, perhaps extending into the hypodermis [53]. The coils are typically about 3.5 mm long, approximately 40 μm in diameter, have a volume close to 0.004 mm^3^[54] and are lined with epithelial cells. A discontinuous layer of myoepithelial cells separates the epithelial cells from the basement membrane [9,53,55]. These are not contractile structures that propel sweat as was once believed [56]. Instead, the myoepithelium provides the structural support that permits the generation of the hydrostatic pressures required to overcome downstream friction and to open the duct pore [57]. Both clear and dark epithelial cells are found in the secretory coils, and it is the former that produces the primary (precursor) sweat [7]. Indeed, sweat is secreted in proportion to the size and neuro-glandular sensitivity of each gland, both of which reveal some plasticity due to changes in habitual sweat gland activation [54,58]. It appears that each secretory coil is surrounded by a capillary cage [59], thereby ensuring an adequate blood supply to each gland and the interstitial space from which the glands extract water and electrolytes.

Downstream from the secretory coil is the distal sweat duct, which is about 75% of the length of the secretory segments [3]. These ducts are relatively straight; they are found in the dermis and are lined with a double layer of cuboidal cells [53,57]. The distal duct is responsible for the active reabsorption of sodium, and the passive reabsorption of chloride and water from primary sweat [7]. Finally, the duct becomes spiral in shape as it traverses the epidermis. Since water continuously moves into and out of these ducts, according to changes in the osmotic potential of the intracellular and interstitial compartments, then bidirectional, transluminal fluxes are always occurring. Therefore, even within inactive sweat glands, which generally contain fluid [7], evaporation from the terminal pore, which contributes to the transepidermal water loss, will concentrate this fluid, resulting in water moving up the concentration gradient to enter the sweat duct [7].

Eccrine sweat glands are identified from ductal pores (puncta) at the skin surface. These have a funnel-like appearance and an inner diameter of about 60–80 μm [3]. Over most of the skin surface, but particularly the non-glabrous regions, these pores are lined with keratinised cells, they are relatively inconspicuous and are located at the intersection of the skin creases [9,52]. These glands participate in temperature regulation. However, for the skin covering the palmar and plantar (glabrous) surfaces of the hands and feet, pores are easily seen along the epidermal ridges [52,60], as perhaps first reported by Grew [17]. These glands are certainly active during thermal sweating [61,62], but they are also powerfully stimulated by various non-thermal influences [3,20].

#### Methodological considerations

##### Gland-counting methods

A range of methods has been used to count sweat pores, and the method chosen can influence the final number of glands observed. In this regard, it is important to first note that not all eccrine sweat glands serve functional roles. That is, the (anatomical) number of glands within a body region invariably exceeds the number of functional or physiologically active sweat glands [63-66], although the proportion of inactive glands varies across studies.

One can determine sweat gland (anatomical) numbers by counting ductal pores. This is performed using skin samples obtained during surgical procedures [67], collected via skin biopsies [68] or harvested from cadavers [69]. It appears that about 5%–10% of these anatomical glands are made up from normally developed, yet inactive sweat glands [65,66].

Physiologically active sweat glands are most frequently identified using colorimetry [70-72] or plastic impression techniques [71,73]. In the former method, glands are identified when sweat interacts with a water-sensitive compound (e.g. iodine, bromophenol blue) painted onto the skin or impregnated into paper which is then applied to the skin [64,74,75]. Colour changes signify the presence and location of secreting pores. For the very sensitive impression technique [76], a polyvinyl solution is applied to the sweating skin. As the rubberised solution dries, sweat droplets form either holes or bubbles within the plastic, marking the presence of a sweat pore. Each of these functional measures relies upon sudomotor activation induced via thermal, non-thermal and pharmacological stimuli. However, there is a significant delay between the first appearance of sweat and the attainment of steady-state gland recruitment and glandular flows [77], so timing the counting of activated sweat glands becomes critical. Moreover, pharmacological stimulation can, in some circumstances, activate more glands than does passive heating [78], particularly if the latter stimulus is only mild, although this is not universally observed [79].

Finally, it is necessary to consider the size of the skin surface from which gland counts are derived since there is density variability not only among sites and individuals, but also within sites from the same subject. Therefore, Weiner and Lourie [80] advocated that gland counting should be from areas as large as 90 cm^2^. This is somewhat unrealistic, particularly for sites such as the fingers and toes, where perhaps even a 2-cm^2^ sample is reaching the limits for single digits. However, the point is still valid, and data from several smaller similar areas can be used to accumulate a suitably large surface area, such that gland counts may reasonably reflect the mean glandular densities for those sites.

##### Sampling affects

Since there is considerable intra- and inter-subject variability in glandular densities, then inadequate consideration of the sample size and subject selection methods can bias research outcomes. Perhaps the most frequently cited data sets for sweat gland counts come from the work of Szabo [67,81]. However, inspection of these reports reveals that both experiments suffered from variable, and in some cases, inadequate sample sizes. For instance, when gland densities were summarised for 11 skin regions in the first report [81], only five or fewer specimens were used to calculate the glandular densities for nine of these regions. In the latter study [67], fresh skin samples were obtained during surgical procedures from approximately 350 donors. This appeared to correct the sample size problem. Unfortunately, due to the method of collecting tissue samples, this problem remained and was compounded by a subject selection problem. Data presented within Table two of that report [67] show that, for the 25 sites for which eccrine gland densities were reported, 13 counts were derived using samples from just one person, and only three sites had samples sizes of ten or more donors. Moreover, these tissue samples were generally not taken from the same individuals. Thus, variations in gland counts among different skin regions with such small samples sizes can yield little meaningful information.

Many studies suffer from these design limitations, so labouring this point serves no additional function. However, in the following section, data from such studies will be used since there is no reason to doubt their precision, but these glandular densities will be weighted to avoid bias introduced through variations in sample size.

#### Inter- and intra-regional variations in sweat gland density

Our focus now turns to examining regional differences in eccrine sweat gland densities, and this analysis was performed against the above background and by merging data from studies spanning 168 years; from Krause [23] to Amano et al*.*[82]. Over these years, six anatomical and 32 physiological studies were identified, with each contributing data to the current derivation of sweat gland densities. Sample sizes varied widely among studies, as did the number of data sets from which data were obtained for each skin region. The preliminary interest was upon obtaining data for all skin surfaces. However, these data were subsequently distilled into 14 regions that were thought to provide the most relevant breadth and depth of information for readers. Thus, for the anatomical studies, a total of 126 data sets were extracted across these 14 sites, with the number of data sets for each site ranging from 2 to 24 and containing from 1 to 32 individuals. For the physiological studies, 323 data sets were identified, with site-specific data set numbers ranging from 4 to 53 and sample sizes varying from 1 to 300. Data from men and women were combined, but only adult samples were included within the current analyses.

**Figure 2:**
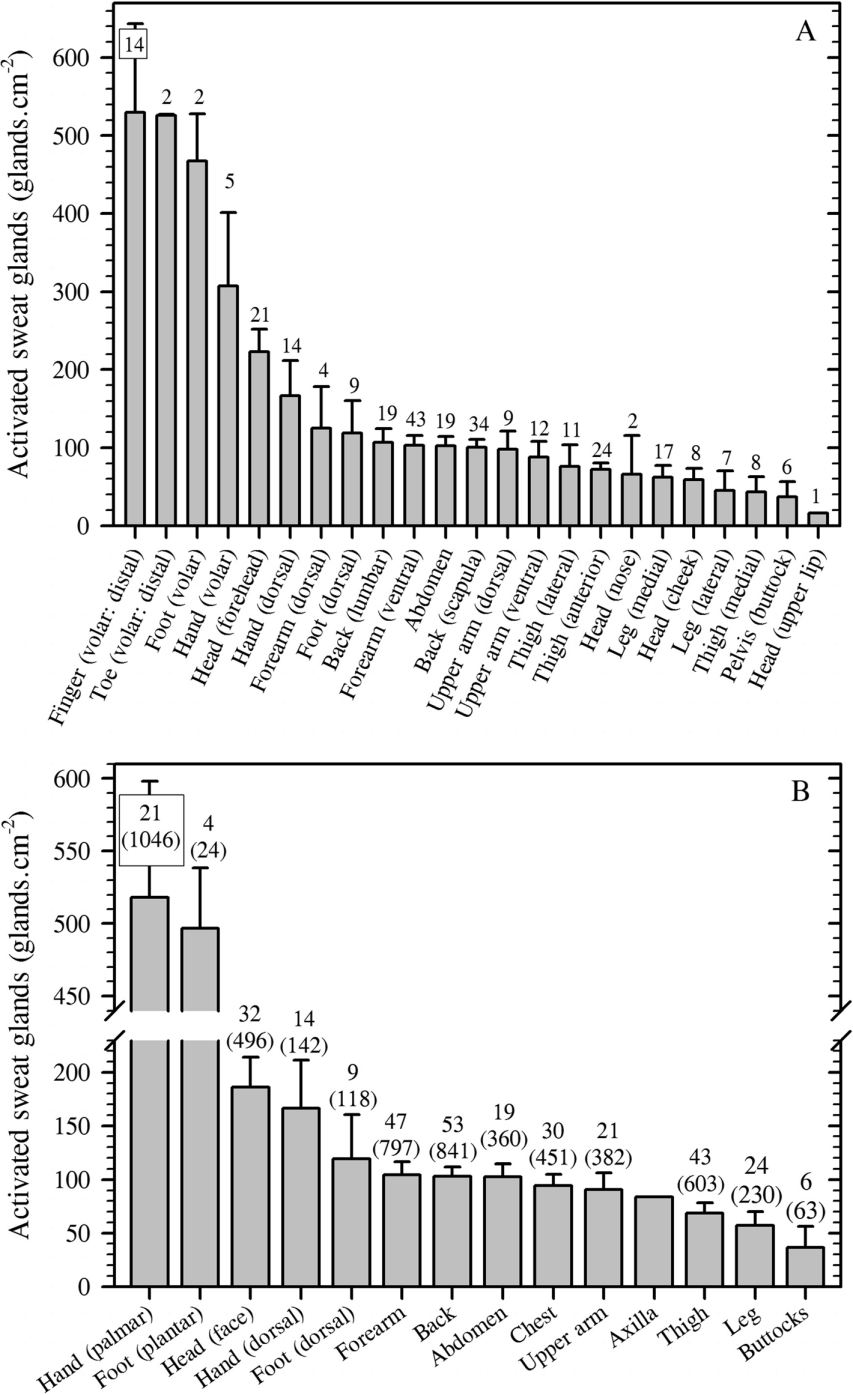
**The distribution of physiologically active, eccrine sweat glands in humans.** Data are means (with 95% confidence intervals) derived across 31 studies, with an average sample size of 23 individuals per data set. The *numbers* above each bar indicate data sets used for that site, with the site-specific sample sizes in parenthesis (**B**). Means were computed as follows: regional density = ((*N*_1_ × density_1_) + (*N*_2_ × density_2_) + … (*N*_i_ × density_i_)) / *N*_Total_ (where *N* is the sample size and subscript numerals refer to separate studies). (**A**) shows all sites investigated. These data were subsequently grouped into 14 larger regions (**B**). The active sweat glands for one region (axilla) have not been investigated, so this value was taken to be 90% of that reported in Table 1. Sources: Clark and Lhamon [60], Ogata [63], Randall [64], Thompson [65], Willis et al. [66], Roberts et al. [78], Sato and Dobson [85], Inoue et al. [86], Kondo et al. [87], Peter and Wyndham [88], Buono [89], Gibson and Shelley [90], MacKinnon [91] (with the original calculation error corrected), Collins et al., [92], Hellon and Lind [93], Silver et al. [94], Ojikutu [95], Sargent and Weinman [96], Juniper and Dykman [97], Toda [98], Bar-Or et al. [99], Knip [100], Knip [101], Schaefer et al. [102], Catania et al. [103], Behm et al. [104], Inoue et al. [105], Kondo et al. [106], Inoue et al. [107], Welch et al. [108] and Madeira et al. [109].

For completeness, data are provided for both anatomical and physiological (active) gland counts. However, discrepancies exist between these numbers, sometimes resulting in more active than inactive glands being identified. This obviously incorrect outcome is because researchers have restricted their interests to either the histological or the physiological domain, and did not perform both measurements. Furthermore, different individuals were investigated across studies, invalidating direct comparisons of these data. In Table 1, the (anatomical) distribution of human eccrine sweat glands is presented for all sites described in the literature. Figure 2A contains data from 24 sites for active sweat glands recruited during thermal, exercise, psychological and pharmacological stimulations. In these studies, fewer sites were investigated, but these active glands are our central focus. Moreover, since most of the interest in gland distribution and sweat secretion centres upon body segments rather than sites within segments, these data were also grouped into 14 regions (Figure 2B). For this grouping, data from Figure 2A were combined to produce segmental averages, and these same 14 regions are emphasised within the subsequent sections of this communication.

**Table 1:** **The anatomical distribution (alphabetical order) and densities of human eccrine sweat glands (glands.cm**^**−2**^**)**

**Sites**	**Gland density**	**Data sets (*N*)**
Abdomen (non-specific)	141 (37)	3 (40)
Abdomen (umbilicus)	82 (21)	2 (40)
Axilla	93 (22)	4 (37)
Back (lumbar)	132 (110)	2 (2)
Back (scapula)	106 (43)	4 (43)
Chest (breast)	21 (14)	2 (34)
Chest (non-specific)	91 (50)	6 (43)
Chest (sternal)	88 (25)	2 (32)
Finger (dorsal: distal phalanx)	126 (75)	2 (28)
Finger (dorsal: middle phalanx)	259 (44)	2 (33)
Finger (dorsal: proximal phalanx)	261 (20)	2 (39)
Finger (volar: distal phalanx)	350 (5)	2 (21)
Foot (dorsal)	155 (59)	6 (54)
Foot (volar: sole)	294 (151)	5 (41)
Forearm (dorsal)	108 (45)	4 (41)
Forearm (ventral)	159 (54)	8 (74)
Hand (dorsal)	176 (79)	5 (46)
Hand (volar: palm)	241 (115)	4 (24)
Head (cheek)	113 (116)	4 (53)
Head (chin)	122 (56)	5 (51)
Head (ear)	140 (−−−)	1 (10)
Head (eyebrow)	61 (16)	4 (43)
Head (eyelid)	190 (−−−)	1 (1)
Head (forehead)	155 (78)	2 (2)
Head (nose)	155 (−−−)	1 (1)
Head (scalp: hairy surface)	195 (58)	4 (48)
Head (scalp: non-hairy surface)	70 (−−−)	1 (1)
Head (upper lip)	132 (40)	3 (53)
Leg (lateral)	115 (51)	3 (44)
Leg (medial)	114 (37)	5 (53)
Neck (non-specific)	126 (109)	5 (39)
Pelvis (buttock)	112 (41)	5 (50)
Pelvis (pubic)	113 (56)	4 (43)
Pelvic (scrotum)	46 (67)	2 (2)
Thigh (anterior)	122 (39)	4 (62)
Thigh (lateral)	102 (18)	3 (46)
Thigh (medial)	89 (−−−)	1 (1)
Thigh (posterior)	31 (−−−)	1 (1)
Toe (volar: distal phalanx)	540 (−−−)	1 (1)
Upper arm (dorsal)	102 (45)	3 (45)
Upper arm (ventral)	94 (42)	5 (50)

Data are means derived from six studies with confidence intervals derived across studies (95% (in parenthesis)) when counts were obtained from more than one data set. For most sites, many data sets were used, and these are indicated in the rightmost column, with the combined sample size for each site in parenthesis. About 5%–10% of these glands are physiologically inactive [65,66]. Sources: Krause [23], Szabo [67], Garcia et al. [68], Hwang and Baik [69], Glaser [83] and Cauna [84]. Calculations: Glandular density for each region was derived as follows: regional density = ((*N*_1_ × density_1_) + (*N*_2_ × density_2_) + … (*N*_i_ × density_i_)) / *N*_Total_ (where *N* is the sample size and subscript numerals refer to separate studies).

It is clear from these analyses that the volar surfaces of the hands and feet have much greater densities of active sweat glands, with four sites from these areas possessing >300 glands.cm^−2^ (Figure 2A). Indeed, these surfaces account for only 5.2% of the total body surface area [110], yet they possess approximately 25% of the total number of sweat glands. Clearly, these glands serve functions beyond temperature regulation. They are known to respond powerfully to changes in emotional and anxiety states [2,15,20,111], and secretion from these surfaces increases both tactile and thermal sensitivity [112,113] whilst simultaneously increasing contact friction and grip [114]. Furthermore, the moisture content of the epidermis acts to reduce the probability of acute tissue damage at these more vulnerable sites [115].

These variations in glandular density exist among individuals due to differences in the number of sweat glands determined during embryonic development, and also due to differences in the eventual body surface area attained during adulthood. Furthermore, within individuals, regional variations in this density are created by divergences in segmental growth. However, the derivation of whole-body, physiologically active gland counts was considered to be a useful teleological exercise, while determining regional gland counts was essential to subsequently computing regional variations in sweat gland output (flow). To perform these calculations, the latest evidence for the relative contributions for each body segment to the whole-body surface area was used [110] in combination with the unisexual, morphological reference adult (phantom: 70.0 kg, 1.702 m, body surface area 1.807 m^2^[116]). The resulting gland counts are presented in Table 2. Since these active gland densities are scaled to the reference adult and since the number of eccrine sweat glands is determined *in utero*, then it is possible to extrapolate these data to individuals with different body surface areas.

**Table 2:** Regional distributions of physiologically active eccrine sweat glands, skin surface areas, gland counts and glandular dimensions

**Site**	**Density (glands.cm**^**−2**^**)**	**Surface area (%)**	**Surface area (cm**^**2**^**)**	**Gland count**	**Gland mass (g)**	**Gland length (m)**
Head	186	7.43	1,342.5	250,021	8.8	1,531.4
Hand (palm)	518	1.81	327.0	169,478	5.9	1,038.1
Hand (dorsal)	166	2.83	511.4	85,153	3.0	521.6
Forearm	104	5.98	1,080.5	112,836	3.9	691.1
Upper arm	91	8.22	1,485.2	134,858	4.7	826.0
Axilla	84	1.09	196.2	16,472	0.6	100.9
Chest	94	7.60	1,374.0	129,742	4.5	794.7
Abdomen	102	7.47	1,349.7	138,315	4.8	847.2
Back	103	12.42	2,244.1	231,408	8.1	1,417.4
Buttocks	37	5.09	919.7	33,751	1.2	206.7
Thigh	69	19.86	3,588.4	246,994	8.6	1,512.8
Leg	57	13.66	2,468.2	141,299	4.9	865.5
Foot (sole)	497	2.90	524.0	260,354	9.1	1,594.7
Foot (dorsal)	119	3.64	657.7	78,272	2.7	479.4
Totals			18,070.2	2,028,954	71.0	12,427

All calculations are normalised to the unisexual, morphological reference adult (70.0 kg, 1.702 m, body surface area 1.807 m^2^[116]). Relative surface areas were obtained from Yu et al. [110], with absolute hand and foot areas from Yu and Tu [117] and Hsu and Yu [118]. Assumptions: The chest and abdomen were assumed to be 50% of the anterior trunk; the anterior neck was added to the chest; each axilla was estimated to be 60% of palmar size, and this was subtracted from the chest; the posterior neck was added to the back. Calculations: Glandular mass calculations were based upon a mean mass of 35 μg [7]. Glandular length (6.1 mm) was based upon a secretory coil length of 3.5 mm long [54] and the assumption that the downstream duct is 75% of this length [3].

The number of eccrine sweat glands found within human skin is reported to range between two and four million [3,23,67,119]. The width of this range is associated not only with natural variation, but also with some investigators studying only anatomical structures, whilst others counted only functional glands. Other causes relate to experimental design limitations such as small sample sizes, combining data from different skin surfaces obtained using different subjects and from not investigating enough skin surfaces. In these cases, approximations of whole-body gland counts suffer from bias. It is hoped that the current approach alleviates this problem, and that from these analyses, one can have considerable confidence in now suggesting that the skin of a standardised individual (1.8 m^2^) will have some 2.03 million functional glands (95% confidence interval 1.72–2.34 million). With each of these glands having a mass of about 35 μg [7], a volume of 0.004 mm^3^[54] and a length of approximately 6.1 mm [3,54], then the combined size of these structures becomes quite impressive (71.0 g, 8.1 cm^3^), as does their total length (12.5 km).

### Regional variations in sweat secretion

#### An overview of eccrine sweat gland function

Human eccrine sweat glands are sympathetically innervated, and it is a curious anomaly that the neurotransmitter for these glands is acetylcholine. However, the sudomotor neurons develop as noradrenergic pathways prior to their postnatal growth and development. At some point after birth, presumably when they first come into close association with their eccrine targets, the neurons change phenotype, becoming cholinergic in nature [120,121]. Indeed, the sweat glands themselves initiate this by producing a chemical trigger for this conversion [122].

Thermal sweating occurs in response to changes in body temperature, with centrally and peripherally located thermoreceptors providing feedback to the preoptic anterior hypothalamus [123,124], which, in turn, activates the eccrine sweat glands. These sympathetic neurons release acetylcholine from their presynaptic terminals, which enters the sweat gland via intercellular canaliculi [125], and stimulates the muscarinic (subtype M_3_) receptors of the clear cells. As a consequence, these cells experience an influx of calcium ions that initiates the pumping of sodium ions into these cells, and through electric coupling, chloride ions also enter [7,125,126]. Water from the interstitial space now follows in an obligatory fashion, as dictated by osmosis [127]. When the osmotic gradient across the basal membranes is removed, water influx ceases. Sodium-potassium pumps on the luminal membrane are activated when an intracellular concentration threshold is achieved, and the active transport of sodium ions, along with chloride and water, into the glandular lumen commences [125]. This fluid is the primary or precursor sweat.

However, altered affective states have long been known to elicit precursor sweat production without the need for changes in body temperature [128-130]. This non-thermal (psychological) sweating was thought, until recently, to be restricted to the glabrous skin of the hands and feet [3,14,131]. Indeed, it was even believed to be induced via separate efferent pathways [132-134] that were of the noradrenergic phenotype [135-138]. However, recent research has established that psychological sweating is a whole-body phenomenon [15,20]. Moreover, the functional relevance of these putative noradrenergic pathways in the control of human eccrine sweating has recently been challenged [21,139]. This research was based on a systemic cholinergic blockade, in combination with whole-body thermal clamping, to eliminate all sweating from the glabrous and non-glabrous surfaces during thermal and non-thermal (psychological and static exercise) stimulations [21]. Accordingly, the assumption from this point onwards is that neurally mediated thermal and non-thermal sweating is of a cholinergic origin. Whilst systemic adrenaline secretion can also stimulate sweating [140], this form of humoral sweat gland activation will not be addressed.

The accumulation of precursor sweat within the duct generates an intra-luminal pressure that forces sweat to flow. Schulz [127] measured these pressures, following the electrophoresis of pilocarpine, by determining the pressure required to halt sweat flow. Intra-luminal pressures increased with flow, with values >65 kPa being observed when glandular flows exceeded 10 μg.min^−1^[127]. The myoepithelial cells provide the necessary structural support for the duct so that these pressures can be generated and an effective sweat flow can be achieved [57].

The composition of precursor sweat is almost identical to that of the interstitial fluid, but the eccrine glands actively reabsorb some elements [141] and thereby participate in regulating the plasma volume and its osmolality. This electrolyte reabsorption occurs within the coiled duct and appears to be highly correlated with the rate of precursor sweat production [85]. Moreover, this solute conservation promotes evaporation at the skin surface, since a more dilute sweat will have a greater water vapour pressure at the same skin temperature [32,142]. However, during extended heat exposures, an accumulation of salt on the skin suppresses water vapour pressure and evaporation, but it simultaneously enhances skin wetting, leading to the formation of a sweat film rather than sweat droplets, a condition which is also more conducive to evaporation [32,142].

Reabsorption is essentially a reversal of the steps for sweat production, albeit now through a much less permeable membrane [126,143]. This is a necessary feature for sustaining epidermal hydration. However, since precursor sweat is flowing, then the sweat duct transit time will dictate electrolyte reabsorption and the composition of discharged sweat [144-146]. Similarly, the interaction of precursor sweat production and reabsorption (water turnover) dictates glandular flow. Nevertheless, since reabsorption is continuous, discharged sweat is hypotonic to both plasma and the interstitial fluid, regardless of flow. This general principle is true for sodium and chloride, but potassium and calcium concentrations increase at lower sweat flows [7]. With this flow dependency in mind, regional variations in sweat composition will be explored within a subsequent section.

Recently, Ohmi et al. [147] used optical coherence tomography to provide images of the last 300–400 μm of the sweat duct lumen as it distended with the accumulation of sweat and shrunk with water reabsorption. From these data, it appears that, at least following psychogenic stimuli, water can remain within the ductal lumen for 150–200 s before either being fully absorbed or reaching the skin surface. We now turn our attention to secondary or discharged sweat secretion, which occurs when the rate of precursor sweat formation exceeds the intra-glandular reabsorption of electrolytes and water.

#### Whole-body sweat losses

Humans have a large and widely variable capacity for the active secretion of sweat. For example, whole-body sweat losses in men can exceed 2 L.h^−1^ during competitive sport [148], with rates of 3–4 L.h^−1^ observed during short-duration, high-intensity exercise in the heat [5,149,150]. Indeed, total daily water losses of 10–16 L have been found during extended physical work performed under stressful climatic conditions [16,151]. However, secretion variations among well-hydrated individuals exposed to the same stress generally reflect age and gender differences [86,152-154], as well as altered sudomotor plasticity associated with exercise- and heat-induced adaptation [155,156], but they are not believed to be due to racial differences [157-159].

Within individuals, it has long been known that much variation exists within whole-body sweat rates across days, and this can exceed the variations observed within a day [160]. Sweat secretion is generally greater in the evening than in the morning [161,162], but this is not reflected within altered sweat gland recruitment or glandular secretion [163]. Moreover, a considerable range of flows may be simultaneously observed across different body regions [164,165]. Therefore, the aim of this section is to provide a comprehensive and contemporary summary of these data, much of which has only recently been described in detail. Before embarking upon this, it is necessary to first describe and critique the techniques used to measure sweat secretion.

#### Methodological considerations

One must remember that sweat appearing on the skin represents water turnover. That is, discharged sweat is the difference between that produced in the secretory coil (precursor sweat) and that reabsorbed within the sweat duct [77,166]. Thus, the first methodological distinction occurs between methods that detect primary (precursor) sweat secretion and those measuring discharged sweat. These are sometimes called internal and external sweating [3,147].

##### Measuring precursor sweat secretion

Bullard [77] quantified the phase delay between sudomotor stimulation and the first appearance of sweat on the skin surface. This delay is attributable to ductal reabsorption, which continues until the surrounding interstitial space becomes congested. When this occurs, a steady-state secretion of sweat will be seen on the skin surface for a given level of sympathetic activity. Thus, in resting, thermoneutral conditions, sympathetic discharge may not elicit measurable sweating since sweat reabsorption may match its formation rate. Under these conditions, sweating is subliminal [167], but it is still of autonomic origin. This was elegantly revealed by Ogawa and Bullard [168] using local pharmacological stimulation to activate sweat glands from two different skin surfaces, both of which then displayed secretion synchronicity, even though subjects were thermoneutral and were not overtly sweating.

One can detect basal, precursor sweat production through changes in skin conductance [169,170], and this galvanic technique is a cornerstone for some psychological stress measurements [171]. For instance, following both non-thermal (e.g. psychogenic) and thermal stimuli, precursor sweat production increases, with the resulting ductal sweat behaving like parallel conductive channels once production exceeds absorption [172]. Indeed, skin conductance increases within just a few seconds [173], and if sweat production at this rate continues, surface (discharged) sweat will soon appear. Of course, reabsorption will also act to elevate interstitial hydration and conductance, and the delay between precursor sweat formation and its first appearance on the skin surface can approach 260 s during mild, passive heating [166]. However, while the galvanic method is very sensitive, and its use is absolutely necessary to convincingly demonstrate an absence or suppression of sweating [21], it does experience saturation at relatively low sweat flows. Thus, it is unsuitable for the quantification of sweat rates. However, a recent advance on this method uses dynamic, optical coherence tomography to measure precursor sweat secretion within individual glands, based upon changes in sweat duct diameter [147]. Unfortunately, this technique requires very sophisticated facilities, and it too is unsuitable for high flows.

##### Measuring discharged sweat

Once sweat reaches the skin surface, it can be seen (qualitative procedures), or captured and quantified. Visual detection was first used over 300 years ago [17] and has been applied extensively [174,175], particularly for identifying active sweat glands. These techniques have even included catheterising individual sweat ducts [176]. However, visual methods are of little use for measuring sweat flows. While serial photographic methods would appear to increase this possibility [3,154], the limitations of this technique were identified long ago [177]. Therefore, these largely qualitative indices have been superseded by techniques that rely upon collecting sweat (gravimetry) or its water vapour (hygrometry).

Sanctorius [30] first used gravimetry to measure transepidermal water loss [12]. This remains the preferred method for determining whole-body sweat rate [178], once corrections are made for mass changes associated with transepidermal water loss, cellular respiration, food and water ingestion, and the voiding of body wastes. This principle has also been used to quantify regional sweat secretion, but now with sweat collected below mineral oil [179], in plastic bags, pouches or within capsules sealed around or over the target region [111,180,181]. In other methods, sweat is collected in filter papers [182] or in absorbent patches sealed to the skin surface [183-185]. Sweat rates are then derived from the mass of sweat collected, or from mass changes of the filter papers or patches.

These volumetric methods are suited primarily to steady-state conditions, as their temporal resolution prevents the tracking of rapid changes in secretion [186]. Furthermore, through the very act of measuring sweat rate, these techniques can modify the flow of sweat onto the skin surface (reactive error), as the vapour pressure close to the skin progressively rises as moisture accumulates on the skin. At higher sweat flows, evaporation is impaired due to the boundary layer air rapidly approaching saturation, with the skin gradually becoming completely wet. Epidermal congestion ensues, although the stratum corneum is capable of absorbing about five times its own mass in water [187]. Not surprisingly, these collection methods can sometimes result in a progressive occlusion of sweat ducts and sweat suppression (hidromeiosis [188-190]), particularly if air flow is prevented for an extended duration. However, these methods will also elevate local skin temperature, and this would tend to enhance both sweat secretion and its evaporation, although this is not always observed [191]. Nevertheless, skin wettedness can be reduced and the evaporative efficiency supported by frequently removing sweat collecting vessels, or changing the filter papers and patches. Indeed, it has been claimed that absorbents with a high moisture retention can also reduce this hidromeiotic effect [186,192]. However, these gravimetric methods seem not to have been simultaneously validated against other methods, although Boisvert et al. [193] observed a significant positive correlation between sweat rates measured using closed sweat pouches and ventilated sweat capsules. Nonetheless, this only seemed valid after moderate, steady-state sweating had been established since the former failed to track sweat capsule data during the first and last 20 min of exercise. Accordingly, one might expect that sweat flows measured via the gravimetric techniques may sometimes under-represent steady-state flows from the naked skin, although recent comparisons of data from two laboratories, obtained using different subjects and techniques, would indicate that this error can be minimised [194]. Nevertheless, across laboratories, one may perhaps consider that such data may better represent sweat rates that would obtain under clothing, particularly multi-layered ensembles, and may therefore be considered to be of greater utility for such applications.

The water vapour content of a gas may be measured using a range of methods. For instance, one can extract water vapour through its condensation within chilled tubes [44] or its absorption into desiccants (e.g. calcium chloride [2,3]). However, these methods are somewhat slow and can lack precision due to the incomplete removal of water vapour [46]. Since water vapour absorbs infrared radiation and also alters the thermal conductivity of a dry gas, then it is possible to determine the water vapour content of an air sample through changes in its infrared light absorption [195] or its thermal conductivity [196]. Nevertheless, neither of these techniques has become popular. Others adopted the approach of quantifying evaporative heat exchange from changes within the water vapour pressure gradient of the boundary layer air [197,198]. While this technique has a broad application, it is not well suited to high sweat rates.

The contemporary hygrometric methods of choice for mechanistic research, where precision in both timing and quantification are required, rely upon the effect of water vapour on electrical resistance [199-201] and capacitance [202,203], or on the dew point of the gas sample [204,205]. Of these methods, capacitance hygrometry seems superior since capacitors are linear across a broad humidity range, and they possess a faster response time when water vapour in the air sample is decreasing [202]. Nevertheless, for each of these techniques, capsules of varying size (e.g. 1–20 cm^2^) are sealed over the chosen skin region [2,46]. To avoid pressure artefacts, an adhesive should be used to make an air-tight seal (e.g. collodion). Air at room temperature, and with a constant and low humidity (often dry gas), is pumped into the capsule and across the skin surface at a fixed flow. This flow is regulated to sustain a dry skin surface (forced evaporation) and thereby optimises the operating range of the hygrometer so that it matches the anticipated local sweat rate. The humidity and temperature of the effluent air are then measured either within or at some point downstream of the sweat capsule [203-205].

These procedures keep the layer of air next to the skin dry and constantly moving, and this not only facilitates transepidermal water loss, but also increases evaporation. This may amplify local sweating (reactive error), relative to that which may have been observed from the naked skin [206]. Using this technique, Hertzman [207] showed that whilst secretion from some sites (calf and some parts of the thigh) exceeded the area-weighted, whole-body sweating (mass loss), sweat flows from other sites (chest and abdomen) were <55% of the whole-body response. In fact, computations of total sweat rate from regional measures generally exceed mass changes [208]. However, the skin below a capsule may be slightly cooler than the adjacent skin surfaces due to greater local evaporation, if the latter is measured without air movement. This can suppress local secretion. Thus, like the patch technique, some localised influences may encourage, whilst others may subdue sweating. Nevertheless, Kenefick et al. [209] recently demonstrated that ventilated sweat capsules have minimal impact upon measurement variation. Therefore, on balance, one may reasonably assume that, while the sweat patch technique is perhaps closer to the fully clothed state, sweat capsules using flows of 500 mL.min^−1^ approximate naked skin exposed to calm conditions (wind speed <1 km.h^−1^).

Missing from this discussion is experimental evidence relating to regional differences in evaporative heat loss. Readers will know that evaporation does not always match local sweat rates, and whilst the perspective presented within this contribution is focussed upon mechanisms that modulate sweating and its regional variations, there is a gap in our knowledge relating to local evaporation rates. During thermoneutral rest, the data presented above for transepidermal water loss (Figure 1), in combination with the specific latent heat of vaporisation, permit one to compute local heat losses. In addition, Park and Tamura [45] presented resting data for subjects exposed to 37°C (women) that may similarly be used. However, for exercising states, local heat losses remain unknown. Since one cannot reliably determine whole-body sweat rates from indiscriminately chosen local sweat flows, then it would be equally imprecise to approximate whole-body evaporative heat loss from either local sweating or evaporative rates.

#### Inter- and intra-regional variations in sweat secretion

A wide range of pharmacological and non-thermal influences can stimulate active sweat secretion, but our focus is limited to thermal sweating. Since glandular densities vary across body regions, then if each gland possessed an identical secretion rate, one could assume that inter-regional variations in discharged sweat would be a simple function of eccrine gland density. However, during passive thermal and exercise stimulations, eccrine glands from different regions discharge sweat at vastly different rates, both across and within individuals [65,85]. For instance, Sato and Dobson [85] reported inter-individual, maximal glandular sweat flows ranging from 4 to 34 μg.gland^−1^.min^−1^, with regional flows of 15.8 μg.gland^−1^.min^−1^ (forehead), 11.5 μg.gland^−1^.min^−1^ (forearm) and 17.9 μg.gland^−1^.min^−1^ (back). From this evidence, it is apparent that regional variations in secretion can be ascribed to both anatomical and physiological variations.

##### The recruitment of sweat glands

When active thermal sweating commences, it generally does so through the low-level and gradual recruitment of eccrine glands [175,177], and subsequently through elevated glandular flows [64,87,177]. During isometric exercise, for example, the activation of silent sweat glands is the principal means through which intensity-dependent increases in sweat secretion from both the glabrous and non-glabrous skin surfaces are achieved [82]. However, some glands do not remain constantly active within a region. Indeed, while the number of active glands may be increasing with thermal loading, these are not always the same glands [3,175,210], with some even decreasing their activity over time [88].

This general recruitment pattern was illustrated by Buono [89] in exercising subjects across six sites, with each displaying a gradual, yet variable, elevation in the number of activated sweat glands as core temperature climbed. This recruitment pattern seems not to be affected by ageing, with glandular flows being reduced [211] whilst the number of activated sweat glands appears to remain constant. However, Kondo et al. [87] have shown these activation stages vary among skin regions during exercise. For instance, at lower-intensity exercise (50% of maximal aerobic power), sweating on the forehead, back and forearm was elevated more through glandular recruitment than through increments in flow, while for the chest and thigh, the opposite was observed. Moreover, at a greater exercise intensity (65%), the chest and thigh experienced a continued activation of these glands, whilst secretion from the other regions depended more heavily upon increasing glandular flows.

When the timing of sweat gland recruitment was compared across skin regions in resting, heated individuals, Kuno [2,3] reported a simultaneous glandular activation from all regions except the palmar and plantar surfaces. Kuno [2] referred to Oehler [212], who is believed to have been first to claim, following visual inspection, that glandular recruitment progressed over the body surface. However, Kuno and his associates [3] found no evidence for a recruitment pattern other than its ubiquitous and simultaneous appearance, regardless of how thermal loading was applied.

Contemporaneously, List and Peet [132] used colorimetry (painted iodine solution) to record regional sudomotor activation during passive heating (with 0.5–1.0 g acetylsalicyclic acid (Aspirin) administration, then hot liquids and radiant heat). From these qualitative methods, they observed considerable recruitment variability across subjects. It seemed that, in some, sweating commenced on the face (forehead and upper lip), whilst in others, it occurred first at the axillae and inguinal folds. They noted that in most individuals, however, sweating commenced on the face and torso before it appeared on the extremities.

However, Hertzman et al. [213] described a caudal-to-rostral (sympathetic dermatomal) recruitment pattern, and this pattern has been accepted by most researchers as the pattern of sweat gland activation. Nonetheless, close examination of that manuscript reveals that neither sudomotor activation nor sweat gland recruitment was measured. Instead, recruitment was determined from changes in the slopes of curves fitted to data points obtained from trials performed in summer and winter, with each point representing a single trial mean. More than 20 air temperatures were evaluated across 61 trials using 22 participants. Thus, these curves summarised group data, and it is uncertain whether data for different skin regions were obtained from the same individuals. Given the wide inter-individual variability in sweating, it is not unreasonable to suggest that such data are less than ideal for drawing such an interpretation.

In a later experiment [214], starch-iodide papers were positioned over different skin surfaces of an unspecified number of resting (supine) heated subjects. No group data were provided to support the dermatomal recruitment hypothesis. Instead graphs for two individuals that displayed this glandular recruitment pattern were published, along with another for an individual with a different pattern. The same group later provided supporting evidence from two more individuals [215]. Certainly time delays between the dorsal foot surface and the forehead are evident within both papers [214,215], but one struggles to resolve time differences among some sites. Moreover, one might contest that, while the dermatomal recruitment of sweating may indeed occur, the data presented did not provide unequivocal support for that hypothesis. It is perhaps time to revisit this theory, but with careful consideration of the postural and pressure affects on sweating.

**Figure 3:**
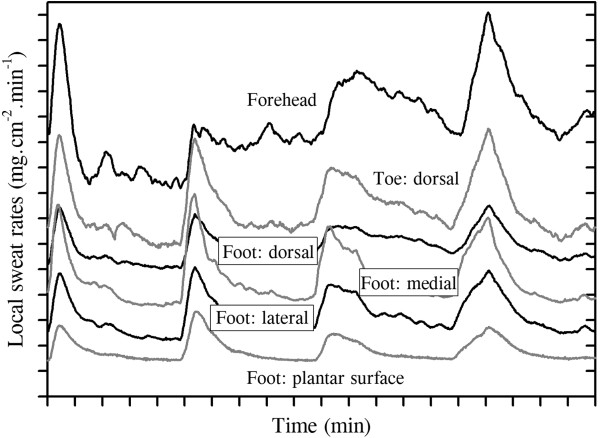
**The synchronous nature of sweating across skin sites.** Sweating during steady-state (passive) thermal loading at rest (air temperature 36°C, water-perfusion suit 40°C). Data are from one individual, collected using ventilated capsules (3.16 cm^2^: forehead, and the dorsal, plantar, medial and lateral surfaces of the foot; 1.40 cm^2^: dorsal toe). *Curves* have been adjusted vertically to reduced overlap and to highlight secretory synchronisation.

Notwithstanding the possibility of a centrally determined sweat recruitment pattern, once activated, a cannulated sweat gland will reveal both a gradually rising column of sweat and a rhythmical rise and fall of this fluid. This was first described by Takahara [216] for individual glands, and was thought to be due to pulsatile contractions of the myoepithelium and induced via changes in sympathetic tone [217]. This rhythm is synchronised within and across body regions [168,200,218,219] and is clearly of autonomic origin, having a period of 0.60–0.74 s [220,221], but it does not result from myoepithelial contraction [57]. This synchrony is illustrated in Figure 3, in which discharged sweat rates were simultaneously recorded from glabrous (hairless) and non-glabrous foot surfaces. Since these data were derived using sweat capsules (3.16 cm^2^), then each curve contains data from many glands. Nevertheless, clear sudomotor synchronisation is evident across all sites. Such synchrony between glabrous and non-glabrous surfaces has previously been described [218], though it is not always evident [222,223], and it demonstrates the existence of neural linkages with the hypothalamus.

##### Inter-regional sweat distributions

There are many studies in which sweat secretion from several sites has been simultaneously measured, and it is from this research that we have extracted data to describe the regional distribution of thermal sweating. To the best of our knowledge, the first such report during resting thermal loading was by Ikeuchi and Kuno [43], while the corresponding quantification during exercising states appears to be that of Weiner [164]. Over the ensuing years, 16 suitable resting studies and 20 exercising studies were identified in which inter-regional variations in sweat secretion were evaluated. These data were pooled for the current analyses. Data from men and women were combined, with only adults being included. A broad range of thermal stimuli were applied, and subjects were tested in different postures (seated, supine). In the exercising studies, both dynamic (arm cranking, bench stepping, cycling, running) and static (handgrip, leg extension) exercise was performed. When data were available across different thermal or exercise conditions, only the more stressful states were selected. Finally, data were collected using various sweat collection techniques (filter papers, sweat capsules, sweat patches). With the exception of exercise states, no effort was made to tease out non-thermal influences. Instead, it was deemed to be more generally useful if integrated analyses were undertaken. Therefore, regional variations for this thermoeffector function, during both exogenous (passive) and endogenous thermal loading, will be described for the same 14 sites used to summarise sweat gland densities. Across all studies and skin sites, the mean resting sweat rate was 0.36 mg.cm^−2^.min^−1^, while the corresponding exercising value was 0.89 mg.cm^−2^.min^−1^. The limitations of such an integrated analysis are widely accepted. However, it was considered that both the depth and breadth of these resources could act to negate many of these drawbacks, and that such an analysis may have broad appeal and application for readers. In the next section, data drawn from experiments conducted using more rigidly controlled experimental conditions and methods are reported and compared with these data.

**Figure 4:**
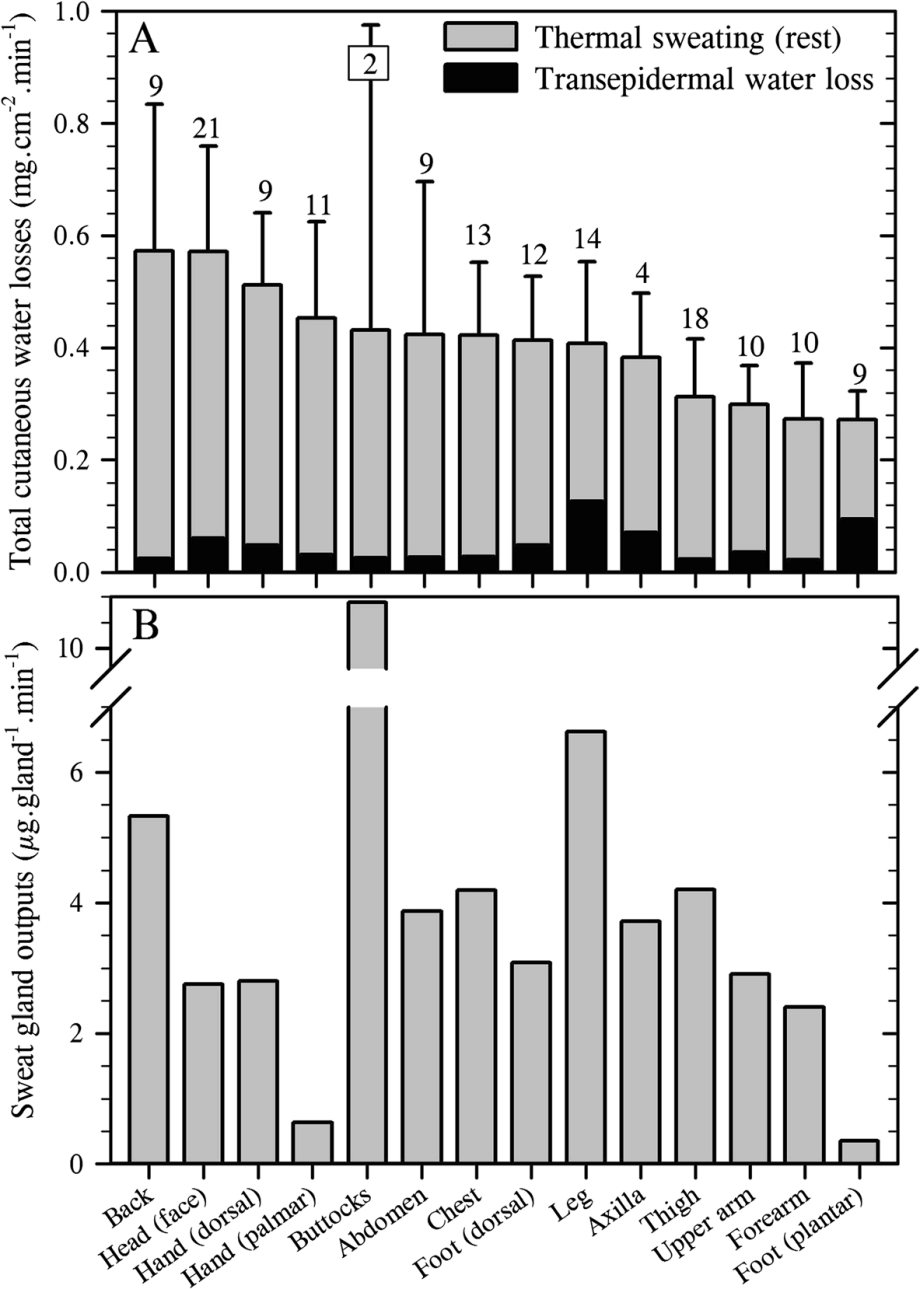
**Total cutaneous water loss and sweat gland outputs in resting, passively heated individuals.** (**A**) Regional variations in total cutaneous water loss (descending order) and (**B**) sweat gland output (ordered as in **A**). (**A**) is a summation of transepidermal water loss (averaged from Figure 1: dorsal foot was assumed to equal the dorsal hand) and thermoregulatory sweating. Sweat data are means (with 95% confidence intervals across studies) derived from 17 studies (191 subjects: numbers are data sets used for each site), with an average sweat rate across all sites and studies of 0.36 mg.cm^−2^.min^−1^. For simplicity, all anterior surfaces of the head were included within the face. For the limbs, data from all surfaces were combined, while the hands and feet were separated according to their dorsal and volar surfaces, with corresponding data obtained from the fingers and toes included within those surfaces. Data for the buttocks came from only two studies with considerable between-study variability and should be treated cautiously. Site-specific sweat rates were computed as follows: regional sweating = ((*N*_1_ × SR_1_) + (*N*_2_ × SR_2_) + … (*N*_i_ × SR_i_)) ∕ *N*_Total_ (where *N* is the sample size, SR is the sweat rate and subscript numerals refer to separate studies). (**B**) shows variations in sweat gland output, derived by combining data from Figures 2B and 4A. Sources: Machado-Moreira and Taylor [20], Machado-Moreira et al. [21], Ikeuchi and Kuno [43], Burch and Sodeman [44], Park and Tamura [45], Taylor et al. [61], Machado-Moreira et al. [62], Inoue et al. [86], Smith et al. [194], Hertzman [207], Hertzman et al. [213], Machado-Moreira et al. [224], Machado-Moreira et al. [225], Cotter [226], Gordon [227] and Machado-Moreira [228].

For this exercise, the transepidermal water loss data described above were combined with thermal sweating data to provide regional variations in total cutaneous water loss (Figure 4A). In addition, by combining functional gland density data, which were assumed to reflect those recruited during resting and exercising states, regional sweat gland outputs (flows) were computed (Figure 4B). In the current analyses, 151 data sets were used across all sites, providing data from 191 individuals studied at rest, and with the number of region-specific data sets ranging from just two for the buttocks [43,45] through to 21 at the face, which included the forehead, cheeks, chin and upper lip. These extant data clearly support the classical conclusion that discharged sweat flow varies across the skin surface of resting subjects [2]. However, the cause of this variation has not been isolated. For instance, while differences between precursor sweat production and reabsorption within the sweat duct determine discharged secretion, as will regional deviations in glandular density and cholinergic sensitivity, regional variations in these attributes have not yet been explored.

Data from one notable resting study [74] were not included in this analysis since the investigators used a sweat box, from which the neck and head protruded, eliciting considerable bias in torso secretion relative to that of the head. For instance, excluding these data from the complete data set resulted in overall sweating changes of 263% for the chest, 170% for the back and 86% for the head.

Whilst there was considerable variation in the distribution of sweating among studies for physiological and perhaps also some methodological reasons, the consensus from these analyses is that the torso (back) and head (face) surfaces have the highest local sweat rates, whilst sites located on the limbs, particularly the feet (soles), secrete the least sweat during passive thermal stimulation. For the most part, however, sweating appears to be relatively homogeneously distributed.

The volar surfaces of the hands and feet, which have the highest glandular densities, possess the lowest glandular flows during resting thermal stimulation, although these sites clearly respond to passive heating [61,62]. In comparison with the torso sites (chest, back), they have about five times more sweat glands, yet sweat gland output from the torso glands is approximately 7–15 times greater (depending upon which sites are compared). Indeed, there is a clear variability in the regional distribution of sweat gland output. For most sites, this pattern is consistent with their local sweat secretion (Figure 4). However, the face produces 4–7 times greater glandular flow relative to the palms and soles, but it has only half the glandular density.

These differences reflect variations in contributions to heat dissipation. If one assumes 100% evaporation, which is not unreasonable in dry heat [31,32], then the back, thighs and legs dominate heat loss at rest. In fact, at secretion rates presented in Figure 4, these sites account for almost 50% of the resting, whole-body evaporative potential (Table 3) due to the combined influences of local surface areas and glandular flows. Indeed, when the torso sites are combined, their collective contribution approximates 40% of the whole-body evaporative heat loss. Furthermore, the site-specific contributions to this heat loss are largely a function of each local surface area, and within the range of variation that one may expect when comparing data across experiments (10%). This generalisation appears somewhat paradoxical when one considers the hands and feet, since their volar surfaces have very low sweat gland outputs. Thus, their particularly high activated glandular densities appear to compensate for these low flows, permitting their potential heat loss to be proportional to their surface areas, at least when resting. This observation is of significance when one considers the thermolytic potential of these appendages.

**Table 3:** Regional contributions to evaporative heat loss (assuming 100% evaporation) from 14 body regions during thermal loading

**Site**	**Rest heat loss (W)**	**Relative contribution (%)**	**Exercise heat loss (W)**	**Relative contribution (%)**
Head (face)	27.81	10.2	91.30	13.8
Hand (palm)	4.33	1.6	12.88	1.9
Hand (dorsal)	9.63	23.5	27.36	4.1
Forearm	10.97	4.0	40.58	6.1
Upper arm	15.87	5.8	36.44	5.5
Axilla	2.48	0.9	6.75	1.0
Chest	22.00	8.1	50.60	7.7
Abdomen	21.71	8.0	45.68	6.9
Back	49.88	18.3	113.12	17.1
Buttocks	14.90	5.5	20.59	3.1
Thigh	42.06	15.4	102.59	15.5
Leg	37.89	13.9	88.56	13.4
Foot (sole)	3.75	1.4	6.83	1.0
Foot (dorsal)	9.75	3.6	17.43	2.6

The mean, whole-body sweat rates under these conditions would approximate 0.4 L.h^−1^ (rest) and 1.0 L.h^−1^ (light-moderate intensity exercise). Calculations were performed using the regional sweat rates from Figures 4 (resting states) and 5 (exercising states), an assumed heat loss of 2.43 kJ.mL^−1^ and the surface area of each region (Table 1) based upon the morphological reference adult [116].

During exercise, endogenous heat production increases, as does the demand for evaporative cooling, and non-thermal stimuli will now affect both sudomotor and vasomotor functions. It is possible that neural feedforward (central command), which emanates from the rostral brain and simultaneously activates the motor and sympathetic neurons, elevates the sensitivity of the sweating mechanism [229,230]. Therefore, in heated resting humans, the initiation of exercise is accompanied by reduced skin blood flow and increased sweating [231,232]. The question of interest now centres upon whether or not the distribution of sweating observed at rest is retained during exercise.

**Figure 5:**
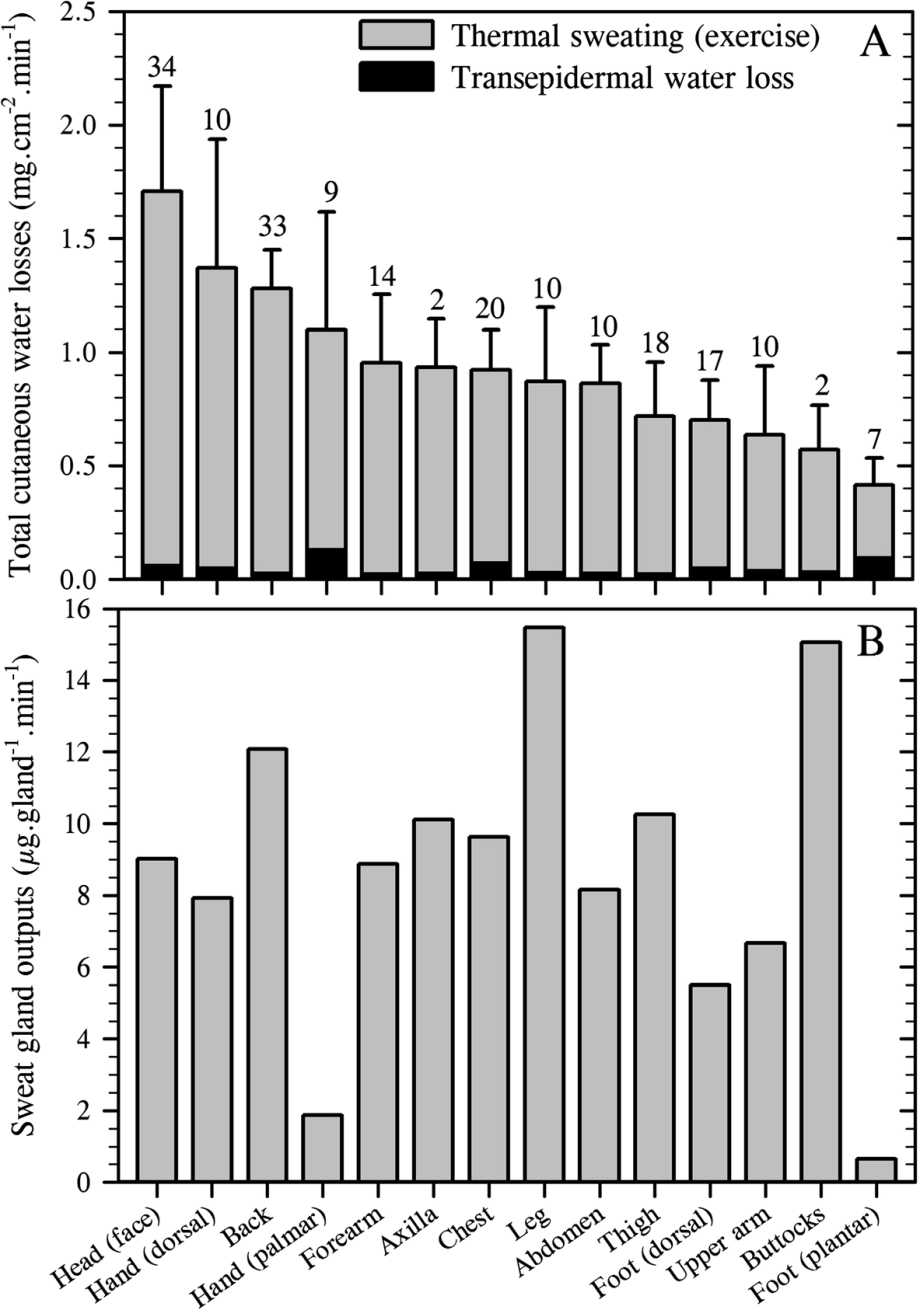
**Total cutaneous water loss and sweat gland output during dynamic and static exercise.** (**A**) Regional variations in total cutaneous water loss (descending order) and (**B**) sweat gland output (ordered as in **A**). (**A**) is a summation of transepidermal water loss (averaged from Figure 1: dorsal foot was assumed to equal the dorsal hand) and thermal and non-thermal sweating. Data are means (with 95% confidence intervals across studies) derived from 20 studies (214 subjects: numbers are data sets used for each site), with an average sweat rate across all sites and studies of 0.89 mg.cm^−2^.min^−1^. For simplicity, all anterior surfaces of the head were included within the face. For the limbs, data from all surfaces were combined, while the hands and feet were separated according to their dorsal and volar surfaces, with data obtained from the fingers and toes included within those surfaces. Data for the buttocks came from only one study. Site-specific sweat rates were computed as follows: regional sweating = ((*N*_1_ × SR_1_) + (*N*_2_ × SR_2_) + … (*N*_i_ × SR_i_)) ∕ *N*_Total_ (where *N* is the sample size, SR is the sweat rate and subscript numerals refer to separate studies). (**B**) shows variations in sweat gland output, derived by combining data from Figures 2B and 5A. Sources: Taylor et al. [61], Machado-Moreira et al. [62], Sato and Dobson [85], Patterson et al. [156], Weiner [164], Patterson et al. [191], Havenith et al. [192], Smith et al. [194], Takano et al. [208], Kondo et al. [87], Smith and Havenith [233], Machado-Moreira et al. [224], Machado-Moreira et al. [225], Gordon [227], Höfler [234], Ayling [235], Cabanac and Brinnel [236], Libert et al. [237], Cotter et al. [238] and Cotter et al. [239].

Weiner [164] described the torso as dominating sweat secretion when exercising in the heat, although his sample was very small (*N* = 3). This conclusion is consistent with the resting data presented in Figure 4 and Table 3, and it also matches, at least qualitatively, the current distillation for exercise which involved 214 different individuals and 196 separate data sets (Figure 5). However, Weiner [164] also suggested that 50% of sweat loss comes from the torso, with a further 25% arising from the lower limbs. Relative to resting data (Figure 4), the absolute sweat flows increase across the body during exercise, but the torso contribution to heat loss (Table 3) declines slightly (40% versus 36%). Nevertheless, sweat loss (Figure 5) again remained proportional to each local surface area (Table 2): head 14%, hand 6%, upper limbs 11%, torso 36%, lower limbs 29% and feet 4%. On this basis, one may conclude that exercise is not associated with a redistribution of sweating, but it is instead accompanied by an almost universal elevation in sweat gland output, such that glandular output becomes more homogeneous across the body surface, even though some regions still have greater secretion rates.

##### Intra-regional sweat distributions

There are inherent limitations to using retrospective data. While the impact of this is reduced as the size of the database grows, this does not generally apply to intra-regional comparisons since the number of data sets available for these sites is very much smaller. Ideally, such data should be drawn from experiments in which variability, due to differences in experimental design, is minimal. In this regard, readers are also directed to the research of Smith and Havenith [233], and these observations were incorporated into the above analysis (Figure 5A). However, due to methodological differences (gravimetric versus hygrometric), they are not included within the analyses below, which focus wholly on data from the authors' laboratory, in which the distribution of sweating was investigated across 45 sites, some of which have not previously been described [61,62,224,225,233].

Steady-state sweat rates were obtained from subjects seated in a climate-controlled chamber set to 36°C (60% relative humidity: no direct air flow onto participants) whilst wearing a whole-body, water-perfusion suit (40°C–46°C). Sweating was measured using ventilated sweat capsules (see ‘Measuring discharged sweat’ section), with data collected at rest (49 subjects across five studies and 45 sites) and during step increments in exercise intensity (cycling: 46 participants across six investigations and 26 skin sites). In the latter studies, trials terminated with core temperatures ranging from 38.9°C to 39.7°C. To avoid a temperature bias, the water-perfusion suit was not in contact with skin surfaces from which local sweating was measured.

**Figure 6:**
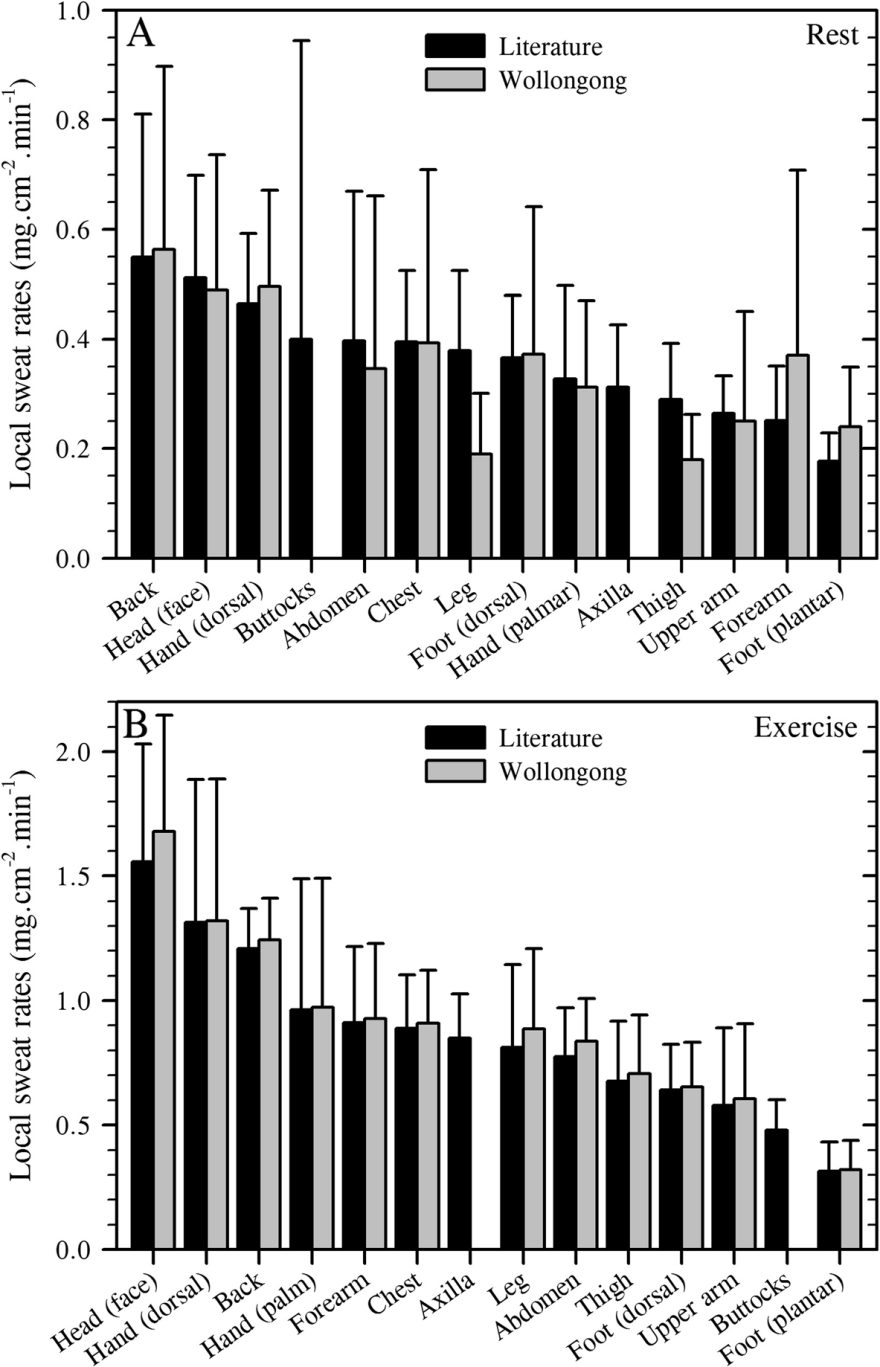
**Comparisons among the regional distributions of eccrine sweating.** (**A**) At rest and (**B**) during exercise. Data are means with 95% confidence intervals. Values distilled from the literature are also contained within Figures 4 and 5 and are duplicated here (in descending order within each graph) for ease of comparison. Data from authors' laboratory (Wollongong: sweat capsules (capacitance hygrometry)) were collected from the studies reported in Figures 7 and 8 but grouped to match the target 14 regions.

To facilitate a first-stage comparison of these data with those reported within the previous section (Figures 4 and 5), site-specific data for the same 14 sites were assembled and presented in Figure 6A (rest) and B (exercise) beside data from the literature. Quantitatively similar outcomes are evident from both analyses, with strong correlations between these data sets (rest: *r* = 0.777; exercise: *r* = 0.996). Whilst data from the authors' research were also included within that obtained from the literature (Figures 4 and 5), such relationships permit greater faith in the retrospective analysis of these data.

**Figure 7:**
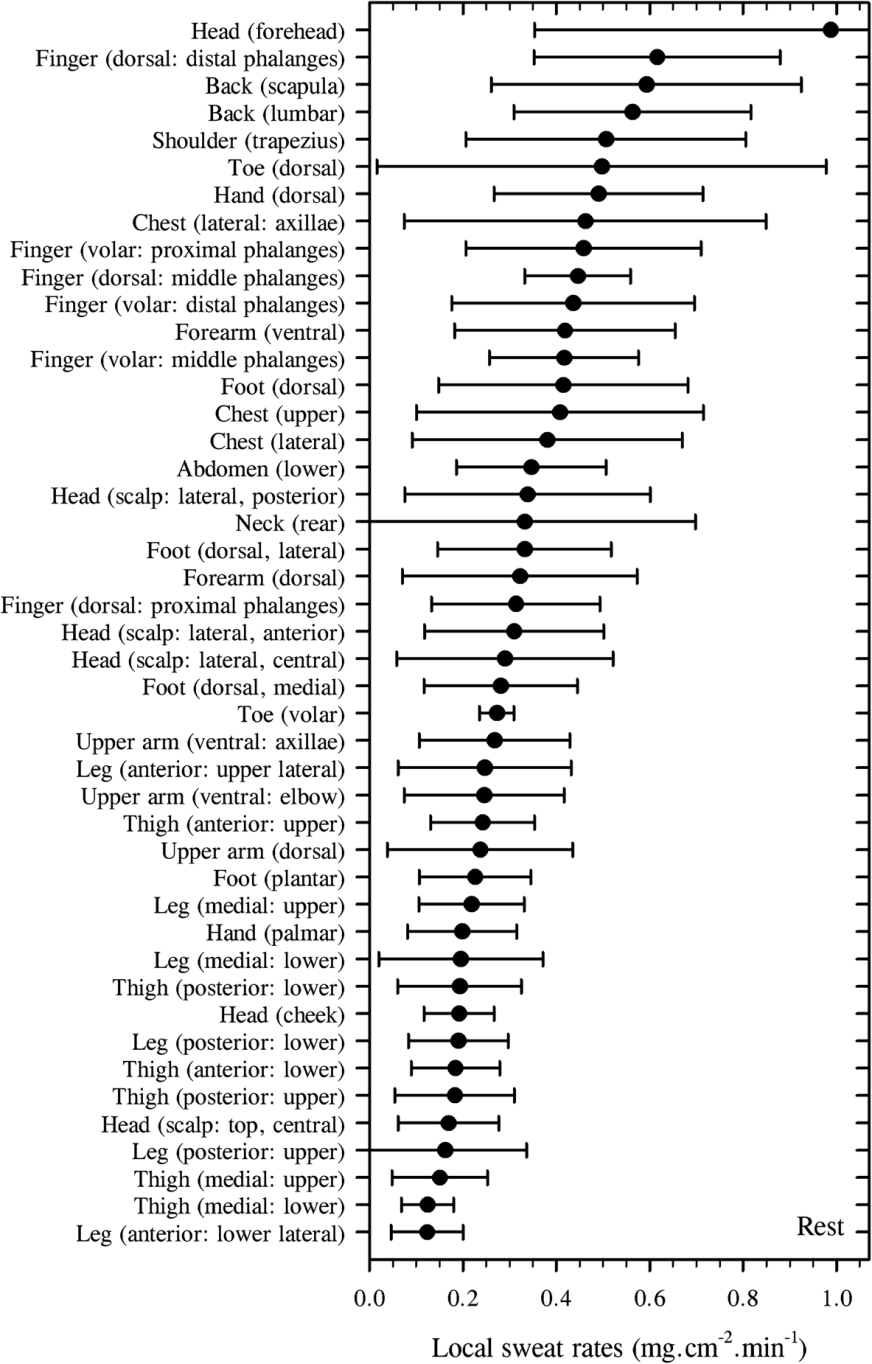
**Inter- and intra-regional distribution of steady-state thermal sweating (ventilated capsules) for resting individuals.** Participants (*N* = 49) were seated (air temperature 36°C, 60% relative humidity) and wore a heated, water-perfusion suit (40°C–46°C). Data are means with standard deviations extracted from five studies undertaken within the authors' laboratory (sweat capsules (capacitance hygrometry)). Sources: Machado-Moreira et al. [62], Smith et al. [194], Machado-Moreira et al. [224], Machado-Moreira et al. [225] and Machado-Moreira [228].

**Figure 8:**
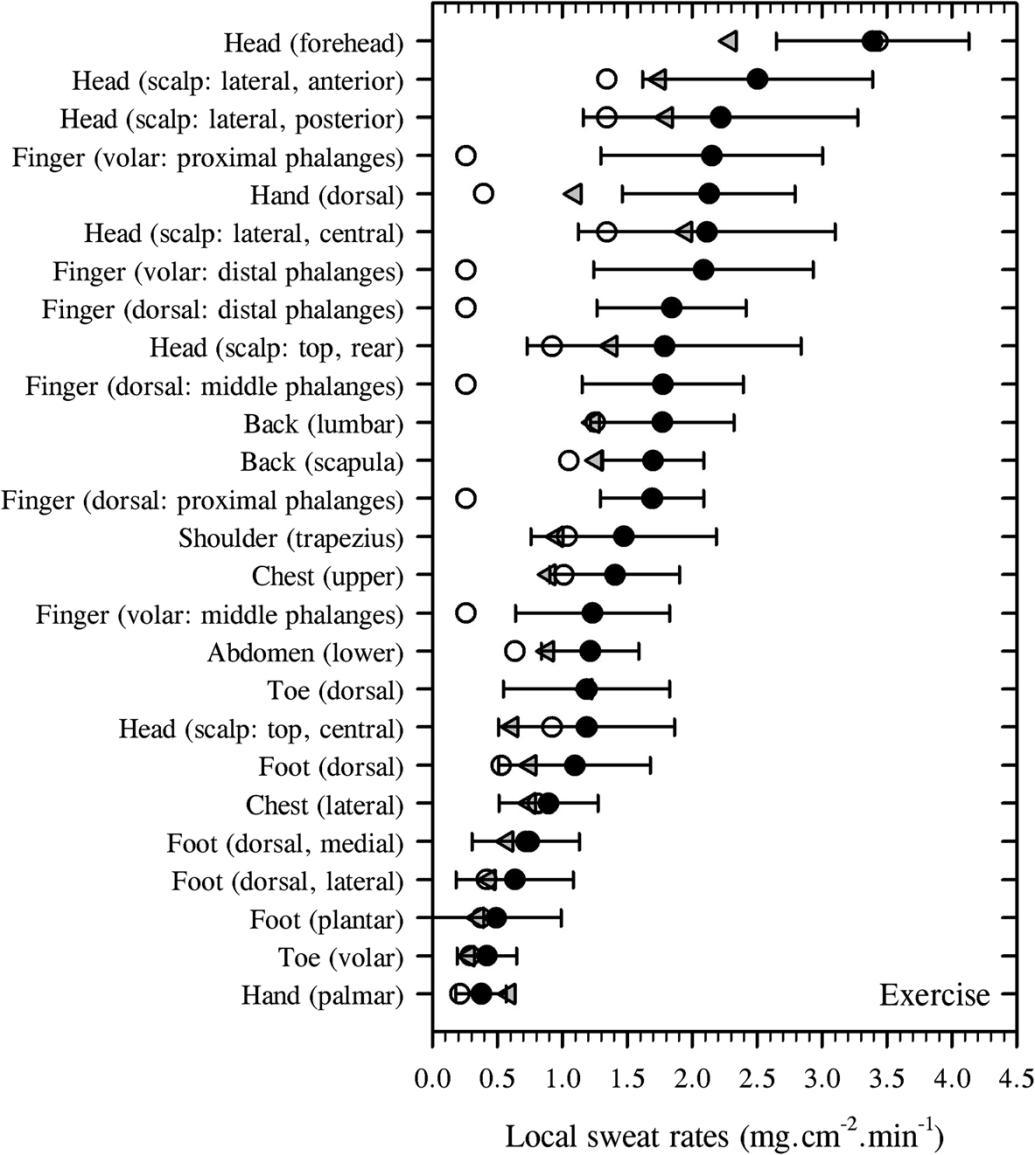
**Inter- and intra-regional distribution of steady-state sweating during exercise.** Data are presented in two forms: (**A**) Mean sweat rates (*closed circles*: ventilated capsules) with standard deviations extracted from six studies undertaken in the authors' laboratory (*N* = 46, cycling: air temperature 36°C, 60% relative humidity: sweat capsules (capacitance hygrometry)). Subjects wore a heated, water-perfusion suit (40°C–46°C). Sources: Taylor et al. [61], Machado-Moreira et al. [62], Smith et al. [194], Machado-Moreira et al. [224], Machado-Moreira et al. [225] and Machado-Moreira [228]. (**B**) For comparative purposes, averages extracted from the literature for exercise (*triangles*) are provided (sources identified in Figure 5). Data reported by Smith and Havenith [233] (second exercise intensity) are also provided (*open circles*).

Having established this broad inter-investigation agreement, we will now present data from our own research that pertain specifically to intra-regional variations in sweating. These data were collected using standardised conditions and methods, and quantify sudomotor variability under both resting (Figure 7: 45 sites) and exercising states (Figure 8: 26 sites). In the latter case, data were averaged across work rates to provide integrated sweat rates at a mean external load of 125 W. However, these data were not collected for limb segments other than the hands and feet. A few subjects participated in several, but not all trials.

Included within Figure 8 are data from two other sources. Firstly, to enable a direct comparison with the comprehensive sweat mapping of Smith and Havenith [233], means from the second exercise intensity of that study (running at 75% of maximal effort; 25°C) have been added (open circles). For some sites (forehead, chest, palm and four foot surfaces), there is strong agreement across these independent studies. Values for another seven sites fall within one standard deviation. However, data for each of the seven hand sites represent considerable under-estimations of the local sweat rates obtained using ventilated capsules, even given the inherent reactive errors associated with each method. Of course, Smith and Havenith [233] studied sweating in somewhat temperate conditions (25°C), so one cannot assume that data from these hand surfaces will reflect changes observed in the heat. Values for the scalp, upper back and abdomen are also lower, but not dramatically so. The third data set presented in Figure 8 comes from the literature (Figure 5: triangles), with sweat rates from only two sites falling beyond the one standard deviation limit (forehead and dorsal hand). One may confidently conclude from Figures 5, 6, 7 and 8 that these data provide similar and valid representations of the distribution of human eccrine sweating.

It is evident from Figures 7 and 8 that the site-specific maximal-to-minimal sweat rates differed by a factor of 8 during resting exposures (forehead 0.988 mg.cm^−2^.min^−1^, anterior surface of the leg 0.124 mg.cm^−2^.min^−1^) and by 9 when exercising (forehead 3.389 mg.cm^−2^.min^−1^, palm 0.375 mg.cm^−2^.min^−1^). These ranges are certainly of practical benefit, but they may also have mechanistic utility. For instance, it has been reported that the volar surfaces of the hand do not participate in thermal sweating [240,241]. Clearly, this interpretation is wrong. Indeed, there are 11 sites that sweat less profusely than the palms during passive heating: four leg, five thigh and two head sites.

In addition, one wonders whether or not there might be a physiological basis for this regional mosaic of sweat secretion, such as a preferential distribution to optimise heat loss efficiency across body segments and surfaces. Since sweating subserves thermal homeostasis, then some insight into this question may be gained from an analysis of the corresponding evaporative potential of each site and combinations of sites, assuming uniform regional skin wettedness, skin temperatures, wind speed and mass transfer coefficients. Of course, these simplifications deviate from reality, but they permit one to consider states within which evaporative heat loss is optimised and modulated primarily by the autonomic control of thermal sweating. Accordingly, these first-principles calculations, whether performed using the regional sweat rates extracted from the 30 investigations reported in Figures 4 and 5, or just the data within Figures 7 and 8, show a remarkable consistency with the size of each surface area represented by these skin regions. For example, of the 14 regions described in Table 2, the five with both the highest evaporative heat loss potential at rest (Table 3) also had the greatest skin surface areas (Table 2). In descending order, these were the back, thigh, leg, head and abdomen. During exercise, the following order was realised: back, thigh, head, leg and chest. From a regulatory perspective, one must conclude that this evidence fails to lend support to an hierarchical configuration of regional sweating in either condition. Indeed, higher secretion rates at any one site would seem merely to reflect that site approaching its full potential for evaporative heat loss for the existing conditions. Furthermore, following heat adaptation, skin regions further away from their site-specific, maximal sweating capacity similarly experience the greatest increase in sweat secretion [156].

With respect to the impact of sweating on the design of clothing and sweating, thermal manikins, the following generalisations may be drawn from the data presented in this section concerning sweat rates observed during exercise. Firstly, for the head, secretion from areas inside the hairline is noticeably lower, being only about 47% of that produced by the facial sites. Secondly, torso sweating is highest on the back, particularly at the lumbar region, followed by the chest and abdomen, with sweat flow from the chest representing about 72% of that produced on the back. Thirdly, the volar surfaces of the hands and feet are thermally responsive, producing 60% and 67% (respectively) of the sweat secreted from their dorsal surfaces. However, both the dorsal and volar aspects of the fingers sweat quite profusely (Figures 7 and 8), while the palms are far less responsive. Finally, concerning limb sweating, secretion from the lower limbs (including the feet) amounts to about 63% of that produced by the upper limbs (including the hands); the upper arms sweat less (68%) than the forearms, while the ratio of thigh-to-leg sweat flow approaches unity (94%).

#### Predicting regional sweat gland densities and secretion rates

The authors assumed that readers would come to this paper from varied backgrounds and interests. With this in mind, the possibility was considered that some, armed only with a knowledge of body surface area, may wish to know eccrine glandular densities for specific regions or site-specific sweat rates during rest and exercise. To address this possibility, Table 4 was constructed, with regression coefficients enabling a first-level prediction of these variables. To illustrate this, prediction equations for the head are highlighted below:

Head sweat gland count = body surface area (cm^2^) × 0.0743 × 186 (glands)

Head sweat rate (rest) = body surface area (cm^2^) × 0.0743 × 0.489 (mg.min^−1^)

(at core temperature of 37.2°C (0.6°C rise) with whole-body sweating approximately 0.4 L.h^−1^)

Head sweat rate (exercise) = body surface area (cm^2^) × 0.0743 × 2.450 (mg.min^−1^)

(at core temperature of 38.8°C (2.2°C rise) with whole-body sweating approximately 1.0 L.h^−1^).

**Table 4:** Coefficients for predicting regional variations in glandular density and sweat rates

**Site**	**Coefficient *A***	**Coefficient *B***		
	**Fractional surface area**	***B***_**1**_**: gland density**	***B***_**2**_**: sweat rate (rest)**	***B***_**3**_**: sweat rate (exercise)**
Head	0.0743	186	0.489	2.450
Hand (palm)	0.0181	518	0.312	1.461
Hand (dorsal)	0.0283	166	0.495	1.851
Forearm	0.0598	104	0.370	0.927
Upper arm	0.0822	91	0.250	0.606
Axilla	0.0109	84	0.312	0.850
Chest	0.0760	94	0.393	1.403
Abdomen	0.0747	102	0.346	1.053
Back	0.1242	103	0.564	1.658
Buttocks	0.0509	37	0.400	0.553
Thigh	0.1986	69	0.179	0.706
Leg	0.1366	57	0.189	0.886
Foot (sole)	0.0290	497	0.240	0.464
Foot (dorsal)	0.0364	119	0.372	0.932

The coefficients can be used to predict regional variations during resting, passive heating and light-moderate exercise. For passive heating, predictions relate to conditions that elicit a core temperature of 37.2°C (or a 0.6°C elevation above baseline), and a whole-body sweat rate of 0.4 L.h^−1^. During exercise, the core temperature was 38.8°C (elevation of 2.2°C), external work rate was 125 W and the sweat rate was approximately 1.0 L.h^−1^. General equation: Body surface area (cm^2^) × coefficient *A* (fractional surface area [110]) × coefficient *B* (site-specific glandular density (*B*_1_: glands.cm^−2^) or sweat rate (*B*_2_ or *B*_3_: mg.min^−1^)). Gland densities are from Figure 2A. Sweat rates are pooled from data in Figures 7 and 8 (authors' laboratory). Data for the axilla and buttocks (in both states), and for the forearm, upper arm, thigh and leg during exercise were obtained from the literature. Bilateral predictions include both limbs.

#### Regional variations in the composition of sweat

Detailed treatment of this topic is beyond the scope of this review, and readers are directed to the literature (e.g. [9,242-244]). However, having described the distribution of eccrine sweat glands and secretion rates during thermal loading, it seems reasonable to include a brief consideration of electrolyte loss since sweat composition [144-146], and therefore cutaneous water vapour pressure [32,142] are influenced by intra-glandular water turnover. Thus, sodium and chloride sweat losses vary in relation to sweat gland output [144-146], but not that of potassium and calcium, which seem to be inversely related to flow [7]. This renders the modelling of sweat composition more complex. Indeed, when one realises that sweat sodium concentrations, for instance, can double in some individuals over the physiological range for sweat production [245-248], then it becomes apparent that quoting sweat compositions without simultaneously reporting glandular or whole-body sweat flow offers little useful information. However, when normalised to sweat flow, sodium losses appear to be independent of gender and can be described using a common flow-dependent relationship (*N* = 12, 36°C, 50% relative humidity, *r*^2^ = 0.94 [248]), at least within young, physically active, unacclimatised individuals:

(1) $$\begin{array}{l} \text{Sweat}\;\text{sodium}\;\text{loss}=-0.674+1.603\\ \;\times \text{sweat}\;\text{rate}\;\left(\text{mg}.{\text{cm}}^{-2}.{\text{min}}^{-1}\right)\\ \;\times \left(g.h^{-1}\right). \end{array}$$

There are many reports that describe the composition of sweat. From these, six were identified that provided both whole-body electrolyte losses and sweat rates [245,249-252]. In some cases, whole-body sweat rates were not provided but could be calculated, and it was found to vary between 0.72 and 3.65 mg.cm^−2^.min^−1^ across studies. Nevertheless, these studies indicate that, when sweating within this zone, the whole-body sodium loss could be expected to fall within the range of 26.5–49.7 mmol.L^−1^ (95% confidence interval), with the corresponding chloride loss being 26.8–36.7 mmol.L^−1^ and that for potassium being 2.7–4.5 mmol.L^−1^. Of course, it is recognised that electrolyte losses are widely variable across [250] and within individuals [191,253].

**Figure 9:**
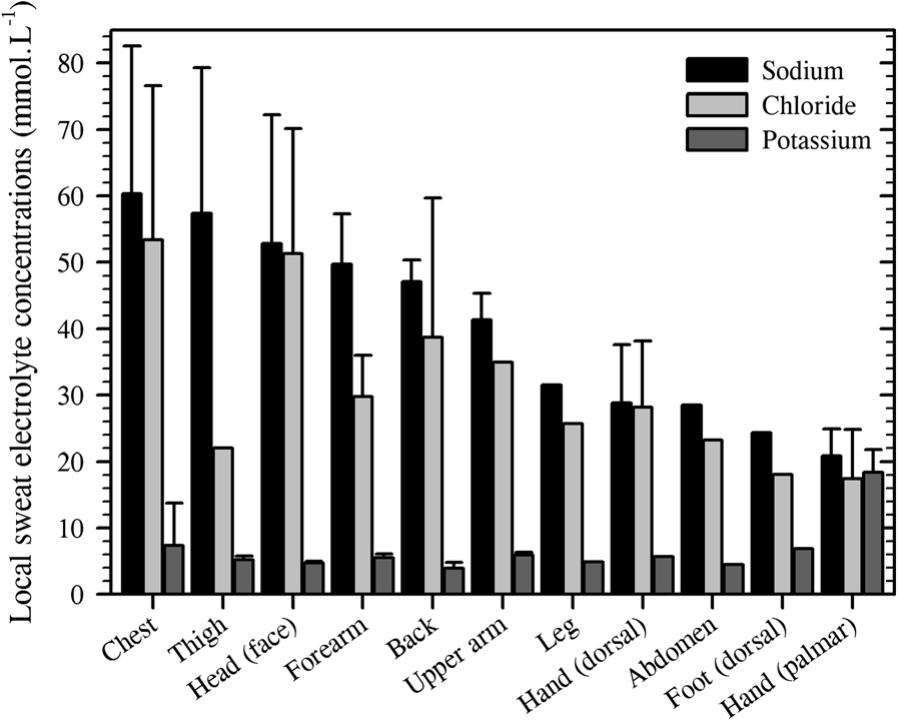
**Regional variations in sweat sodium, chloride and potassium concentrations.** Data were obtained from 13 papers that reported both electrolyte concentrations and sweat rates, or information that permitted derivation of the latter. These sweat rate ranges and sample sizes for each site are provided in Table 5. Data are means with 95% confidence intervals across studies. Site-specific sweat compositions were computed as follows: regional composition = ((*N*_1_ × *X*_1_) + (*N*_2_ × *X*_2_) + … (*N*_i_ × *X*_i_)) ∕ *N*_Total_ (where *N* is the sample size, *X* is the electrolyte composition and subscript numerals refer to separate studies). Sources: Sato et al. [79], Patterson et al. [191], Allan and Wilson [246], van den Heuvel et al. [248], Yousef and Dill [251], Hayden et al. [255], Collins [256], Locke et al. [257], Emrich et al. [258], Costa et al. [259], Cage et al. [260], Boisvert et al. [261], Fukumoto et al. [262] and Saat et al. [263].

Thirteen papers were identified that provided simultaneous sweat secretion and composition data for several of the 14 body regions of interest. These data are summarised in Figure 9 and Table 5 and, with the exception of the thigh, provide a reasonable reflection of regional variations in electrolyte losses over the flows indicated (Table 5). These data tend to over-estimate whole-body electrolyte concentrations and, like local sweat rates, should generally not be used to approximate whole-body losses [249], although some sites do lend themselves to such use [191,254]. Readers need also to be aware that daily variations within individuals can approach 10% [255].

**Table 5:** **Regional sweat secretion ranges (mg.cm**^**−2**^**.min**^**−1**^**) and sample sizes (*N*) for data presented in Figure**9

**Site**	**Sodium**	**Sample**	**Chloride**	**Sample**	**Potassium**	**Sample**
Chest	0.76–3.11	23	1.21–3.11	17	0.76–3.11	23
Thigh	0.66–0.88	31	0.66	10	0.66–0.88	31
Head (face)	2.00–6.00	15	2.00–6.00	15	2.00–6.00	15
Forearm	0.06–1.38	63	0.06–1.38	40	0.06–1.50	71
Back	0.56–3.53	139	0.85–2.90	28	0.85–2.90	71
Upper arm	0.52–0.75	16	0.52	10	0.52–0.57	16
Leg	0.76	10	0.76	10	0.76	10
Hand (dorsal)	0.56–0.91	12	0.21–0.91	23	0.91	8
Abdomen	0.65	10	0.65	10	0.65	10
Foot (dorsal)	0.56	9	0.56	9	0.56	1
Hand (palm)	0.02–0.12	18	0.03–0.12	6	0.02–0.12	18

These data are electrolyte-specific sweat rate ranges and samples sizes for the site-specific concentrations summarised in Figure 9.

The regional differences in electrolyte losses may reflect variations in physiological function. For instance, within any region, one would expect that sweat sodium and chloride concentrations might correlate with local sweat gland output since a slower ductal transit time would permit greater reabsorption. However, to the extent that one may apply data from Figures 4 and 5 to those from Figure 9, this did not obtain. There can be no doubt that, at the glandular level, this does occur. But there were no meaningful correlations across regions between differences in electrolyte concentration and either sweat rate or the glandular output derived at rest and during exercise, as previously reported [191]. Indeed, glandular flows appear to become more homogeneous when exposed to combinations of thermal and exercise stress. Moreover, at the palm, which has a very high activated glandular density but a particularly low glandular flow, sweat sodium and chloride concentrations are at their lowest, whilst potassium loss appears to be more than twice that of any other region (Figure 9). This last observation was derived exclusively from the data of Collins [256], and must be treated cautiously, since the mean is three-four times larger than the plasma concentration of potassium. This could indicate water absorption into the stratum corneum [249] or electrolyte leaching associated with the method of sweat collection [264]. Notwithstanding possible errors, our current understanding of the physiology of eccrine glands does not adequately describe these *in vivo* variations.

**Figure 10:**
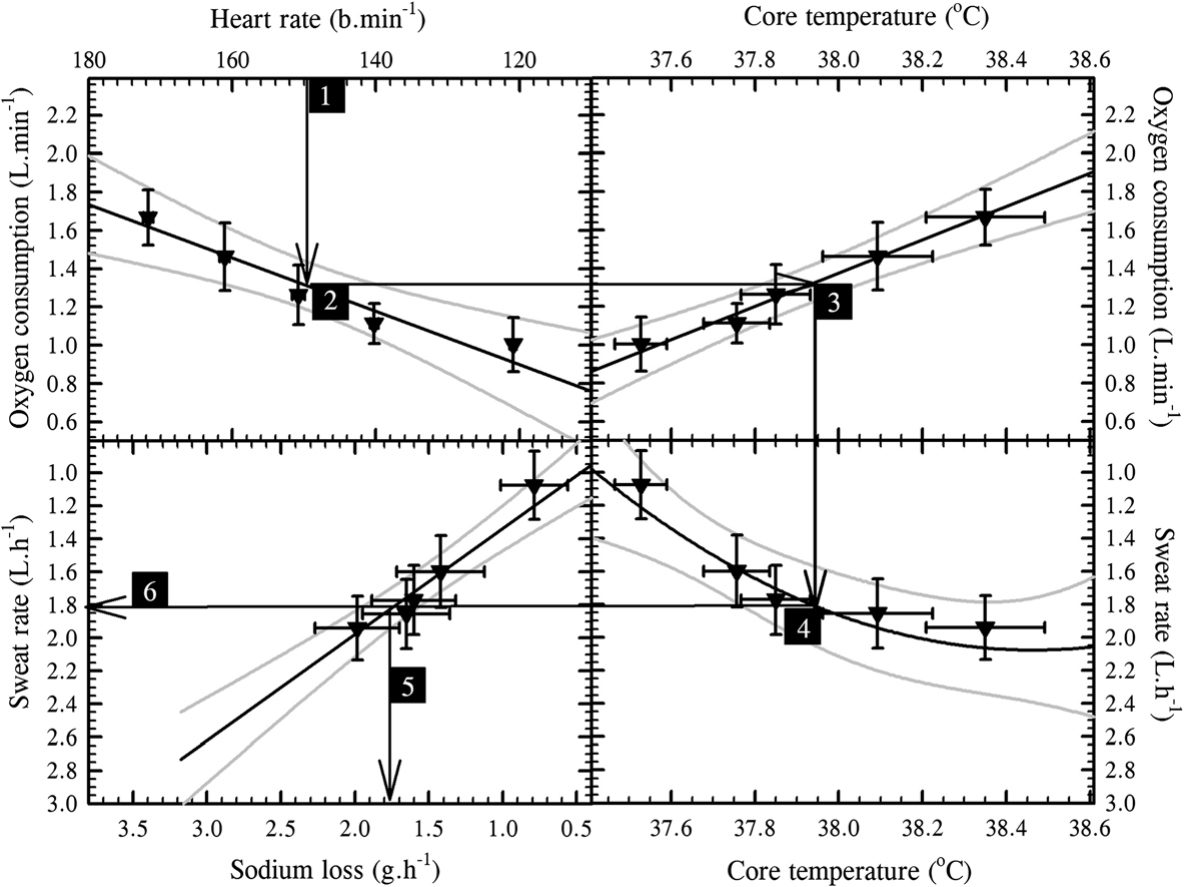
**A quadrant diagram for estimating fluid and sodium replacement rates.** This prediction uses heart rates obtained during steady-state cycling in the heat (36°C, 50% relative humidity). Data were collected from six, fully-hydrated and unacclimatised females [268]. Regressions show 95% confidence intervals. Commencing from *position 1*, enter the quadrant to successively approximate the oxygen consumption (*position 2*), core temperature (*position 3*) and sweat rate (*position 4*) that would obtain for the measured heart rate. Predictions of sodium (*position 5*) and fluid replacement rates (*position 6*) to sustain body-fluid and electrolyte homeostasis can then be approximated.

In keeping with our desire to provide information of pragmatic benefit, we bring this section to a close by answering the following question: From a knowledge of steady-state heart rate alone, could one estimate fluid and sodium requirements during physical activity? Through the use of four well-established relationships, one could indeed arrive at such an approximation, at least within homogeneous population samples. For instance, it is well known that oxygen consumption increases linearly with exercise-induced increments in heart rate [265] and that body core temperature is intrinsically linked with oxygen consumption [266]. Furthermore, sweat rate increases asymptotically with core temperature [267], whilst sweat sodium secretion is a positive linear function of sweat rate [246]. To evaluate the possible utility of these linked relationships, data were collected from six males and six females during steady-state exercise in the heat [248]. Figure 10 summarises the results of the women, presented as a quadrant diagram (adapted from [248]). Whilst these relationships vary widely across the entire population, within homogeneous groups, the predictive precision tightens to the extent that first-level approximations may be derived to support body-fluid and electrolyte homeostasis in the field (see figure caption for details).

#### Conclusions

Regional variations in transepidermal water loss, eccrine sweat gland densities, sweat rates and electrolyte losses have been demonstrated. From these observations, it has been determined that a standardised individual (70 kg, 1.7 m) would possess some 2.03 million functional glands, with the highest density on the volar surface of the finger (532 glands.cm^−2^) and the lowest on the upper lip (16 glands.cm^−2^). Under a resting heat loading, the forehead (0.99 mg.cm^−2^.min^−1^), dorsal fingers (0.62 mg.cm^−2^.min^−1^) and upper back (0.59 mg.cm^−2^.min^−1^) of this person would generally display the highest sweat rates, whilst the medial thighs and anterior legs would secrete the least (both 0.12 mg.cm^−2^.min^−1^). When exercising in the heat, all sweat rates will increase, such that glandular flows will become more homogeneous. Using regional body surface areas and sweat rates obtained across 45 sites within one laboratory, predictions of glandular densities and local sweat rates for 14 skin regions have been derived. These predictions may be useful to modellers and engineers. However, since evaporative heat loss potential was found to be roughly proportional to local skin surface areas during both rest and exercise, then there appeared to be little evidence to support the possibility of an hierarchical distribution of sweating under either state.

## Competing interests

The authors declare that they have no competing interests.

## Authors’ contributions

NAST conceived this project, planned and coordinated data collection, and wrote the manuscript. CAM-M heavily participated in all stages of this project. Indeed, CAM-M performed the necessary laboratory research that not only underpins this review, but which formed a large part of his Doctoral dissertation. Both authors read and approved the final version of this manuscript.
